# Unraveling the Diversity of Characteristic Aroma Components in Huangjin Green Tea Across Different Flavor Categories

**DOI:** 10.3390/foods15152650

**Published:** 2026-07-28

**Authors:** Hao Huang, Yufei He, Xi Zhao, Penghui Yu, Ni Zhong, Bowen Xiang, Jing Ning, Na Li, Huaisheng Huang, Xinggang Zhong, Qincao Chen, Hongfa Zheng

**Affiliations:** 1 Hunan Tea Research Institute, Hunan Academy of Agricultural Sciences, Changsha 410125, China; haohuang_08@163.com (H.H.); 13142263327@126.com (Y.H.);; 2 Yuelushan Laboratory, Changsha 410128, China; 3 Jishou Tea Industry Development Service Center, Jishou 416000, China; 4 College of Agriculture, Jiangxi Agricultural University, Nanchang 330045, China

**Keywords:** Huangjin green tea, aroma types, volatile organic compounds, HS-SPME-GC-MS, relative odor activity value

## Abstract

Originating from Hunan Province, China, Huangjin green tea (HJGT) is known for its diverse aromas, including green, floral, and chestnut-like, but their characteristic volatile components remain unclear. In this study, 111 volatiles were identified from 26 HJGT samples using HS-SPME-GC-MS. Floral HJGT showed the highest relative content of ester, chestnut-like HJGT was richest in sulfur-containing compounds, and green HJGT was dominated by alcohols. Partial least-squares discriminant analysis identified 61 differential volatiles (VIP > 1), whose distribution patterns across aroma types fell into four categories. Based on relative odor activity value (≥1) and ANOVA (*p* < 0.05), 26 key aroma-active compounds were further screened. Ten characteristic compounds, such as linalool, indole, and dimethyl sulfide contributed floral, fresh, green, cabbage, and violet-like notes to floral HJGT. Seven compounds, including heptanal, nonanal, and 2-pentylfuran, imparted nutty, waxy, pungent, and beany notes to chestnut-like HJGT. Nine compounds, including geraniol, hexanal, and phenylethyl alcohol, contributed green, floral, fragrant, and minty notes to green HJGT. These findings elucidate the chemical basis of HJGT aroma and provide a theoretical foundation for the targeted regulation of its aroma types in production.

## 1. Introduction

China is the homeland of tea, with a history of tea cultivation and consumption spanning several thousand years [1]. According to the processing method and fermentation degree, tea is generally divided into six main categories: green tea, black tea, white tea, yellow tea, dark tea, and oolong tea [2]. Among these, green tea, as an unfermented tea, undergoes high-temperature enzyme inactivation (fixation) that rapidly inhibits enzymatic activity, thereby maximizing the retention of natural compounds in the fresh leaves. This process endows green tea with its unique quality characterized by a clear infusion, green leaves, and a fresh, brisk taste [3]. Green tea not only dominates tea consumption in China but has also gained global consumer preference, driven largely by its appealing flavor and well-established health-promoting properties, such as antioxidant and lipid-lowering effects [4,5]. As consumer demand continues to evolve and the tea market becomes increasingly segmented, the flavor quality of green tea has attracted growing attention in both scientific research and industrial practice. Among the various quality attributes, the complex and appealing aroma characteristics have emerged as a particularly important area of interest.

Aroma serves as a key quality parameter for tea and plays a direct role in determining the commercial value of tea products [6]. The aroma profiles of green tea are remarkably diverse, encompassing a broad spectrum of odor types, including chestnut-like, bean-like, floral, fruity, fresh, and tender notes [7]. These distinct aroma types are generally co-determined by specific geographical origins, tea varieties, cultivation practices, and processing techniques [8,9,10,11]. For instance, high-temperature fixation combined with appropriate roasting tends to promote the development of chestnut-like or bean-like aromas [12], whereas lower-temperature, gentler processing methods are more likely to preserve floral or fresh aroma compounds [13]. Recent progress in analytical chemistry and sensomics has greatly facilitated the detailed characterization of the chemical underpinnings of various green tea aroma types, as well as the recognition of their principal odor-active constituents. However, systematic comparative investigations of volatile composition differences and the identification of characteristic aroma components among multiple coexisting aroma types within region-specific, distinctive tea varieties remain an important topic requiring further in-depth exploration.

Huangjincha (HJC) is a locally distinctive tea germplasm originating from Xiangxi Prefecture, Hunan Province, China [14]. This variety is highly valued for its superior economic traits, which include early budding, high productivity, excellent intrinsic quality, and robust environmental adaptability. Originally a wild resource, HJC has undergone systematic selection and propagation and has now become an important pillar for rural revitalization and the upgrading of the tea industry in the Xiangxi region [15]. The free amino acid content in individual HJC plants can reach as high as 7.47%, which is more than double the average level of other tea varieties harvested in the same period, while the contents of polyphenols and caffeine are relatively moderate. Green tea processed from HJC fresh tea leaves exhibits an excellent quality profile characterized as fragrant, green, refreshing, and full-bodied, earning it the reputation that it is “valued as highly as gold” [16,17]. A notable feature of HJC is that green tea processed from its fresh leaves exhibits considerable aroma diversity, with the products falling broadly into three categories: floral (F), chestnut-like (C), and green (G). The coexistence of these three distinct aroma types within the same tea cultivar, produced under comparable processing conditions, offers a valuable natural system for exploring the chemical mechanisms that govern aroma formation.

To investigate the volatile composition differences among the three aroma types of Huangjin green tea (HJGT), headspace solid-phase microextraction coupled with gas chromatography-mass spectrometry (HS-SPME-GC-MS) was employed in this study for the enrichment and identification of volatile compounds in tea samples. This analytical platform is widely used for qualitative and semi-quantitative analysis of tea volatiles [18,19]. The HS-SPME component enables efficient headspace sampling, while GC-MS provides high-resolution separation and reliable mass spectrometric detection, making the combined technique particularly suitable for comprehensive volatile profiling [20]. Furthermore, this study introduces the relative odor activity value (rOAV) method, which integrates compound semi-quantitative values with their odor thresholds (OT), enabling effective discrimination of characteristic components that genuinely contribute to the overall aroma profile, thereby avoiding potential misinterpretation based solely on relative abundance [19,21]. The combined strategy of HS-SPME-GC-MS and rOAV has become a recognized, efficient, and reliable analytical approach in contemporary tea aroma research [22].

The primary objective of this study was to systematically compare the volatile compound profiles of HJGT across its three aroma types (floral, chestnut-like, and green). Candidate marker volatiles discriminating these aroma types were identified through partial least squares discriminant analysis (PLS-DA) combined with variable importance in projection (VIP) scores and further prioritized using rOAV calculations in conjunction with odor descriptive analysis, allowing the determination of putatively characteristic aroma-active components for each aroma type, and that direct sensory validation (GC-O, recombination, omission) will be required in future work. The outcomes of this research are expected to clarify the chemical basis for the formation of the three characteristic aroma types of HJGT, and to offer a theoretical foundation and practical guidance for the targeted modulation of processing parameters, the precision enhancement of aroma quality, and the development of specialty tea products with distinct flavor profiles.

## 2. Materials and Methods

### 2.1. Chemicals and Reagents

Deionized water was prepared using a Milli-Q water purification system (Millipore, Billerica, MA, USA). A standard mixture of n-alkanes (C7–C40, GC grade) was purchased from Sinopharm Chemical Reagent Co., Ltd. (Shanghai, China). Ethyl decanoate (purity ≥ 99.0%), used as the internal standard, was acquired from Sigma-Aldrich (Shanghai, China).

### 2.2. Tea Materials and Sensory Assessment

All HJGT samples were procured from tea markets in Jishou City, Hunan Province, China, between March and April 2025. Initially, the tea evaluation panel gathered and screened seventy green tea samples. Preliminary assessment involved discarding any samples with off-odors, followed by aroma evaluation of the remaining samples. Those exhibiting distinctive aromatic characteristics were selected from all HJGT samples for further analysis. The aroma types of all HJGT samples were independently determined by five trained panelists affiliated with the Tea Research Institute of Hunan Province. All panelists had extensive experience in tea sensory evaluation and performed the assessment following the Chinese national standard methodology for tea sensory evaluation (GB/T 23776-2018) [23]. For each sample, 3 g of tea leaves was infused with 150 mL of boiling water for 4 min. The resulting infusion was then filtered, and the aroma of the infused leaves was evaluated by the panelists. This study was approved for exemption from ethics review under Article 32 of the Measures for Ethical Review of Life Science and Medical Research Involving Human Beings (2023, China).

### 2.3. Extraction of Volatile Compounds

For each sample, 1.0 g of tea was precisely weighed and transferred into a 20 mL headspace vial, followed by the addition of 6 µL of ethyl decanoate (100 mg/L) as the internal standard. Subsequently, a magnetic stir bar and 6 mL of boiling water were added to the vial for extraction. Assuming that the final extraction volume was approximately 6 mL (the volume of added water, without considering the slight volume occupied by the tea solids), and the final concentration of the internal standard in the extract was calculated as 100 μg/L. The vial was then incubated at 60 °C for 10 min without agitation. After this period, the SPME holder was introduced through a low-bleed silicone septum, and the fiber was extended into the headspace for volatile extraction over a period of 60 min. Upon completion of extraction, the fiber was retracted and immediately transferred into the GC-MS injection port, where desorption was carried out for 5 min before chromatographic separation.

### 2.4. GC-MS Analysis

Chromatographic separation was carried out on an Agilent 7890B gas chromatograph coupled with an Agilent 5977A mass spectrometer (Agilent, Santa Clara, CA, USA). Volatile compounds were separated on an HP-5 MS capillary column (30 m × 0.25 mm × 0.25 μm, Agilent, USA). The column oven was programmed as follows: the initial temperature was set at 40 °C and held for 5 min, then increased to 160 °C at 3 °C/min, and finally raised to 250 °C at 10 °C/min, with a final hold of 5 min. The injector temperature was maintained at 250 °C, and helium (>99.999% purity) was used as the carrier gas at a constant flow rate of 1.0 mL/min. All injections were performed in splitless mode.

Mass spectrometric detection was conducted under electron impact (EI) ionization at 70 eV. The ion source and quadrupole temperatures were set to 230 °C and 150 °C, respectively. Mass spectra were recorded in the full-scan mode over a mass-to-charge (*m*/*z*) range of 50 to 550.

### 2.5. Volatile Compounds Identification and Semi-Quantification

Qualitative identification of volatile compounds was performed by matching the acquired mass spectra against the NIST20 spectral database, combined with retention index (RI) values and comparisons with previously reported literature data. The compound showing the highest spectral matching score was tentatively assigned as the putative identity. RI values were then automatically calculated by injecting a series of n-alkanes (C7–C40) under the same chromatographic conditions. Compounds with an RI deviation of less than 20 between the experimentally determined and literature values were retained for subsequent analyses. The RI was calculated using the following Equation (1):

(1) $$\left.\mathrm{RI}\;=\;100n\;+\;100\;\times \;\left(RT_{x}\;-\;RT_{n}\right)/(RT_{n+1}\;-\;RT_{n}\right)$$

RI—the retention index of the target compound;

n—the carbon number of the n-alkane standard eluting immediately prior to the target compound;

RT_x_—the retention time of the target compound;

RT_n_—the retention times of the n-alkanes with n carbon atoms;

RT_n+1_—the retention times of the n-alkanes with n+1 carbon atoms.

For semi-quantification, ethyl decanoate was used as the internal standard. The relative content of each identified volatile compound was calculated from the ratio of its peak area to that of the internal standard [24]. According to the following Equation (2):

(2) Ci = Cis × Ai/Ais

Ci—mass semi-quantitative value (μg/L) of any given component;

Cis—mass concentration of the internal standard (μg/L);

Ai—chromatographic peak area of an individual component;

Ais—chromatographic peak area of the internal standard.

### 2.6. rOAV Analysis

To evaluate the sensory relevance of the detected volatiles, the relative odor activity value (rOAV) was calculated for each compound according to the method described by Xiao et al. [19]. The rOAV serves as a widely used indicator for estimating the potential contribution of individual odorants to the overall aroma profile. Compounds with rOAV > 1 were considered as potential key contributors to the characteristic aroma of the tea samples. rOAV were calculated using the Equation (3):

(3) rOAV = Ci/OTi

rOAV—relative odor activity value of aroma compounds;

Ci—mass semi-quantitative value (μg/L) of any given component;

OTi—odor threshold of the corresponding component in water (μg/L).

### 2.7. Statistical Analysis and Data Visualization

All measurements were carried out in triplicate, and the results were presented as mean values with standard deviations (SD). Multivariate statistical analyses, including unsupervised principal component analysis (PCA) and partial least squares-discriminant analysis (PLS-DA), were performed using SIMCA-P (Version 14.0, Umetrics AB, Umeå, Sweden). To identify volatile compounds with statistically significant differences among the three aroma types, one-way analysis of variance (ANOVA) was conducted using PASW Statistics software (Version 18.0, Chicago, IL, USA) to determine significant differences. Graphical representations were generated using Origin 2021 (OriginLab, Northampton, MA, USA), TBtools-II (Version 2.446, Guangzhou, China), and Adobe Illustrator 2021 (Adobe, San Jose, CA, USA).

## 3. Results and Discussion

### 3.1. Aroma Profile Characterization of HJGT Samples

Following preliminary and secondary sensory evaluations of 70 collected HJGT samples, 26 representative samples covering the three characteristic aroma types were selected for further analysis, including 12 chestnut-like, 8 green, and 6 floral HJGT samples. The sensory evaluation results for the different aroma types of HJGT samples are presented in Supplementary Table S1. The tea samples exhibited distinct aroma profiles, which were categorized into three types: floral, chestnut-like, and green. The sensory panel assigned overall aroma quality scores ranging from 91 to 95, indicating consistently high quality across all samples. Notably, the floral HJGT samples were not confined to a single floral type; instead, they exhibited diverse floral notes, such as orchid-like, honeysuckle-like, and rose-like aromas. The chestnut-like samples could be categorized into two subtypes: chestnut-like and tender chestnut-like aroma. Meanwhile, the green aroma samples were characterized by a delicate fragrance. The samples with defined aroma types were subsequently subjected to HS-SPME-GC-MS analysis.

### 3.2. Analysis of Volatile Compounds in Three Aroma Types of HJGT

**Figure 1:**
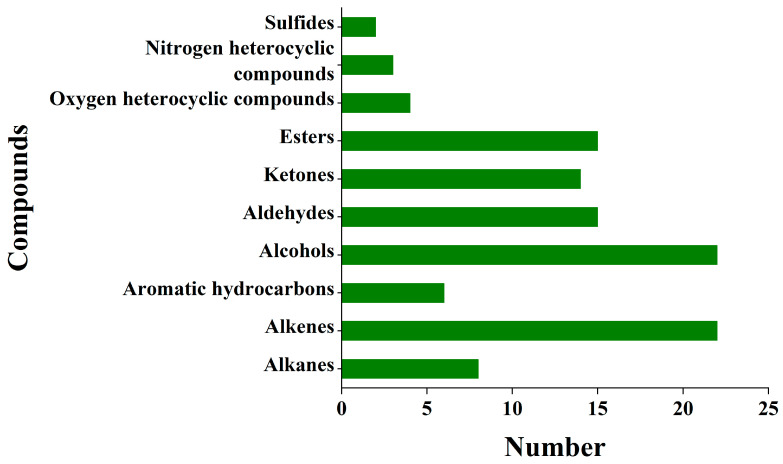
Category and numbers of compounds in HJGT.

Through comprehensive screening by mass spectrometry identification, retention index determination, and multiple complementary methods, a total of 111 shared aroma components were identified (Table S2). Based on differences in their chemical structures, these compounds were classified into ten categories (Figure 1): alkanes, alkenes, aromatic hydrocarbons, alcohols, aldehydes, ketones, esters, oxygen-heterocyclic compounds, nitrogen-heterocyclic compounds, and sulfur-containing compounds. The majority of these volatile compounds have been reported in previous investigations [25]. In terms of compound counts, alcohols and alkenes were the two most predominant classes, each accounting for 22 compounds, followed by aldehydes and esters (15 compounds each), ketones (14 compounds), alkanes (8 compounds), aromatic hydrocarbons (6 compounds), oxygen-heterocyclic compounds (4 compounds), nitrogen-heterocyclic compounds (3 compounds), and sulfur-containing compounds (2 compounds). The importance of alcohols, ketones, and aldehydes as major aroma contributors in green tea, as reported in earlier studies [26], is further supported by the findings of the present work.

**Figure 2:**
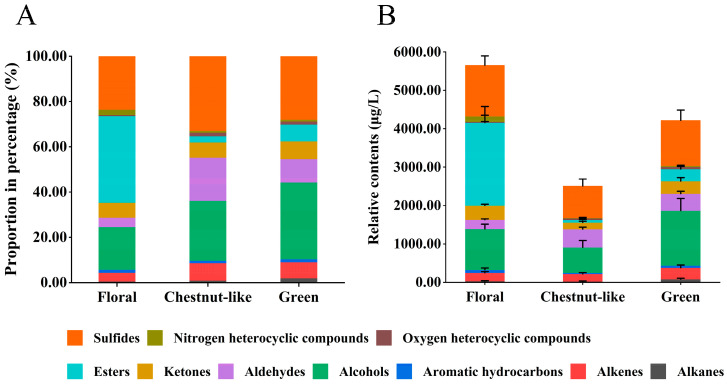
Volatile compound profiles of the three HJGT aroma types: (**A**) relative abundance distribution, and (**B**) relative contents of the identified volatile compounds.

Overall, the three aroma types of HJGT exhibited marked differences in the relative abundance of volatile compounds. The overall volatile abundance was highest in the floral and lowest in the chestnut-like HJGT samples (Figure 2). The present results differ from those reported by Liu et al. [22], a discrepancy that may reflect the use of a unique tea germplasm (HJC) in our study. Furthermore, regional variations in tea products and differences in processing techniques could also be important contributing factors [27]. The volatile fraction of HJGT was dominated by sulfur-containing compounds, with alcohols and esters ranking as the next most abundant classes. Collectively, these three chemical groups represented 72.19% of the total volatile content. Among all volatile compounds identified in HJGT, dimethyl sulfide, (Z)-3-hexenyl hexanoate, linalool, geraniol, 4-methyl-3-penten-2-one, (Z)-3-hexenyl butanoate, (Z)-3-hexen-1-ol, and hexanal were present at relatively high levels, collectively accounting for 64.9% of the total volatile composition. In floral teas, esters constituted the highest proportion (36.3%), followed by sulfur-containing compounds (24.01%). In chestnut-like teas, sulfur-containing compounds were predominant (33.42%), followed by alcohols (27.33%). In green teas, alcohols represented the largest fraction (32.95%), followed by sulfur-containing compounds (27.63%). These results differ from previous reports which suggested that ketones play a dominant role in HJGT aroma [18,28,29]. These inconsistencies are likely attributable to variations in sample preparation and analytical protocols among studies, underscoring the impact of methodological choices on volatile compound determination.

Among the different aroma types of HJGT, the floral aroma HJGT samples exhibited the highest semi-quantitative values of ester compounds, followed by sulfur-containing compounds and alcohols. Subsequently, ketones, aldehydes, and alkenes were detected. Together, these six classes accounted for 95.10% of the total volatile relative content. In the chestnut-like aroma HJGT samples, sulfur-containing compounds showed the highest semi-quantitative values, followed by alcohols and aldehydes. Alkenes, ketones, and esters were present at lower levels, with the six categories collectively comprising 95.69% of the total volatiles. For the green aroma HJGT samples, alcohols were most abundant, followed by sulfur-containing compounds. Aldehydes and ketones ranked next, ahead of esters and alkenes. These six classes together represented 94.58% of the total volatile composition. Overall, among the three aroma types, the floral aroma HJGT samples contained higher levels of esters, the chestnut-like aroma HJGT samples were richer in sulfur-containing compounds, and the green aroma HJGT samples showed a predominance of alcohols. The above results underline the contribution of certain volatiles to the aroma characteristics of HJGT and reveal their differential distribution among the three aroma types [7].

**Figure 3:**
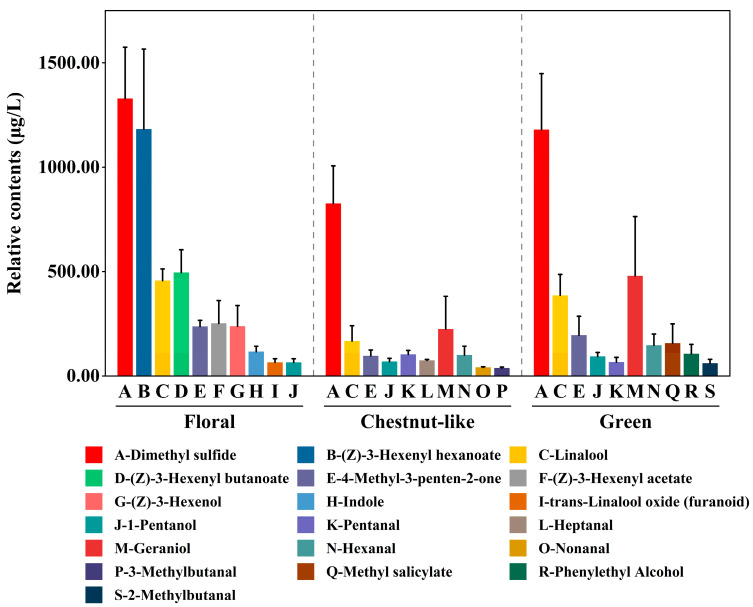
Relative contents of the top 10 volatile compounds in floral, chestnut-like and green HJGT samples.

Therefore, a notable characteristic of the volatile composition in floral HJGT is its abundant relative content of esters, sulfur-containing compounds, alcohols, ketones, aldehydes, and alkenes. Figure 3 presents the top ten volatile compounds and their relative contents in the three types of HJGT. The most abundant components in the floral HJGT, listed in descending order, are dimethyl sulfide, (Z)-3-hexenyl hexanoate, linalool, (Z)-3-hexenyl butanoate, 4-methyl-3-penten-2-one, (Z)-3-hexenyl acetate, (Z)-3-hexenol, and indole. The volatile profile of chestnut-like aroma HJGT is primarily composed of sulfur-containing compounds, alcohols, aldehydes, alkenes, ketones, and esters. Its major constituents include dimethyl sulfide, linalool, pentanal, heptanal, 4-methyl-3-penten-2-one, and geraniol. In green HJGT, the volatile composition is dominated by alcohols, sulfur-containing compounds, aldehydes, and ketones, followed by alkenes and esters. The key compounds, ranked from the highest to the lowest semi-quantitative values, mainly consist of dimethyl sulfide, linalool, 4-methyl-3-penten-2-one, 1-pentanol, pentanal, geraniol, hexanal, methyl salicylate, and phenylethyl alcohol (Figure 3). Notably, four volatile compounds including dimethyl sulfide, linalool, 4-methyl-3-penten-2-one, and 1-pentanol were commonly detected across all three aroma types of green tea. Moreover, our results were partially in line with the findings of Jiao et al. [27], in which linalool, geraniol, and hexanal were likewise among the top ten compounds in terms of semi-quantitative values. These findings indicate that the aroma type differentiation in HJGT is primarily driven by differences in the composition and relative abundance of key volatile compounds.

### 3.3. Multivariate Statistical Analysis of Volatile Compounds in Three Aroma Types of HJGT

**Figure 4:**
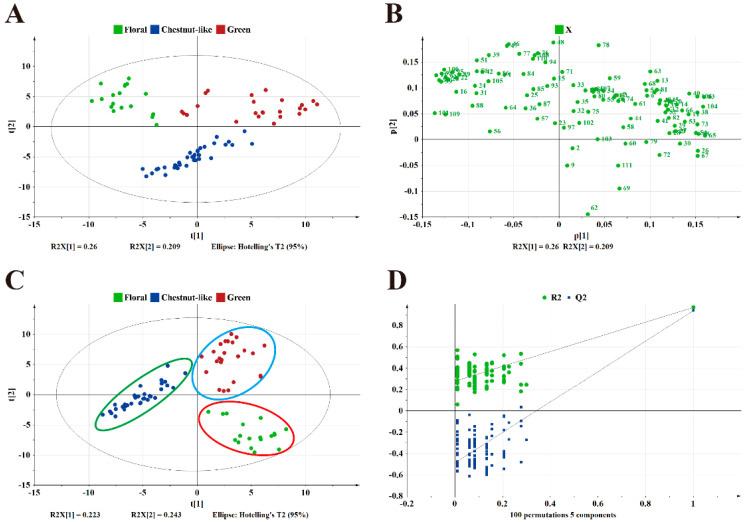
Multivariate statistical analysis of volatile compounds in floral, chestnut-like, and green HJGT samples. (**A**): PCA score plot; (**B**): PCA loading plot (numbered compounds correspond to the IDs listed in Supplementary Table S2); (**C**): PLS-DA score plot; (**D**): PLS-DA model validation by permutation test (200 random permutations).

To further understand the volatile differences among the three aroma types of HJGT, multivariate statistical analysis was performed on the 111 identified compounds. PCA revealed that the first two principal components accounted for 24.8% and 20.7% of the total variance, respectively (R^2^X = 0.813), with all samples falling within the 95% confidence interval, suggesting a satisfactory model fit. The model clearly separated the samples of the three aroma types (Figure 4A), consistent with the predefined classification, which suggests distinct volatile profiles among the different aroma types. Specifically, floral HJGT samples (green dots) were distributed in the upper-left quadrant, green HJGT samples (brown dots) were clustered mainly in the upper-right quadrant despite slight dispersion in a few cases, and all chestnut-like HJGT samples (blue dots) were located below the *X*-axis. The loading plot is presented in Figure 4B.

A supervised PLS-DA model was constructed to pinpoint the volatile compounds most responsible for the discrimination among the three aroma types. This approach enhanced the separation among the groups and facilitated the screening of differential metabolites. The established PLS-DA model (with fit parameters R^2^X = 0.629, R^2^Y = 0.951, and Q^2^ = 0.911) effectively distinguished the chestnut-like, green, and floral HJGT samples (Figure 4C). It further separated green and floral samples into distinct clusters, with all three aroma types clearly distributed across different quadrants, demonstrating significant inter-group differences. The robustness of the PLS-DA model was further evaluated through a permutation test with 200 random permutations (Figure 4D). The intercept values for the permuted models were substantially lower than those of the original model (R^2^ = 0.27, Q^2^ = −0.48), indicating that the model was not overfitted and exhibited satisfactory predictive ability.

**Figure 5:**
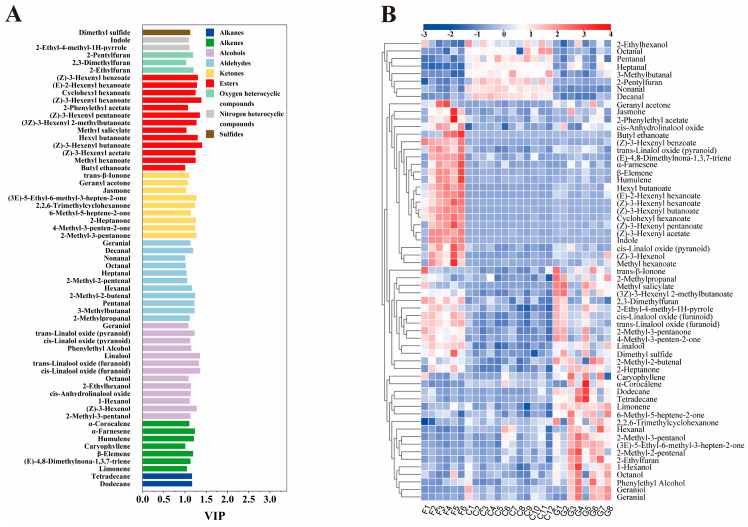
VIP scores (VIP > 1) derived from the PLS-DA model (**A**) and heatmap visualization of the candidate differential volatiles across all HJGT samples (**B**). In the heatmap, red represents high relative content, white indicates intermediate levels, and blue denotes low content.

To rank the relative importance of each volatile compound in differentiating the three HJGT aroma types, VIP scores were derived from the PLS-DA model. Compounds with VIP values greater than 1 were regarded as important discriminators [30,31]. As shown in Figure 5A, 61 candidate differential compounds were screened using the criterion VIP > 1, comprising 13 alcohols, 13 esters, 11 aldehydes, 9 ketones, 7 alkenes, 3 oxygen-heterocyclic compounds, 2 alkanes, 2 nitrogen-heterocyclic compounds, and 1 sulfur-containing compound. A heatmap was constructed to visualize the distribution patterns of the 61 differential compounds across the three HJGT aroma types, based on their semi-quantitative contents (Figure 5B). Four major clusters were identified among the 61 volatile compounds on the basis of their relative content patterns. Among them, 22 compounds, including (Z)-3-hexenyl hexanoate, (Z)-3-hexenyl butanoate, linalool, (Z)-3-hexenyl acetate, (Z)-3-hexenol, indole, (E)-2-hexenyl hexanoate, (E)-4,8-dimethylnona-1,3,7-triene, (Z)-jasmone, and cyclohexyl hexanoate which primarily impart floral, fruity, and grassy notes [32], showed relatively higher relative contents in the floral HJGT. Eight compounds including pentanal, heptanal, nonanal, 3-methylbutanal, decanal, 2-pentylfuran, 2-ethylhexanol, and octanal, which mainly contribute roasted and nutty aromas, exhibited higher relative levels in the chestnut-like HJGT. Meanwhile, 17 compounds such as geraniol, hexanal, phenylethyl alcohol, dodecane, limonene, 2-ethylfuran, tetradecane, and 6-methyl-5-heptene-2-one, which are characterized by grassy and herbal notes, were present at relatively higher semi-quantitative values in the green HJGT. Furthermore, compared with the chestnut-like group, 14 compounds including dimethyl sulfide, methyl salicylate, 4-methyl-3-penten-2-one, trans-linalool oxide (furanoid), 2-methyl-3-pentanone, cis-linalool oxide (furanoid), (3Z)-3-hexenyl 2-methylbutanoate, and trans-β-ionone, which convey fruity and grassy aromas, showed higher relative contents in both the floral and green HJGT samples. However, the sensory relevance of these candidate compounds remains to be corroborated through targeted validation experiments.

### 3.4. Calculation of rOAV for Volatile Compounds in the Three HJGT Aroma Types

It is well recognized that only a limited number of volatile compounds detected in food matrices actually contribute to the overall aroma characteristics. Only a small proportion of compounds confer distinct aromatic notes and play a major role in shaping the characteristic flavor. Such volatile components are referred to as key aroma compounds or odor-active compounds [33]. To further screen for key aroma contributors in each HJGT aroma type, rOAVs were calculated for the volatile compounds identified by GC-MS, using their published odor thresholds in water as reference. Compounds with rOAV > 1 were considered to make a meaningful contribution to the overall tea aroma. A higher rOAV corresponds to a greater contribution [34,35].

**Figure 6:**
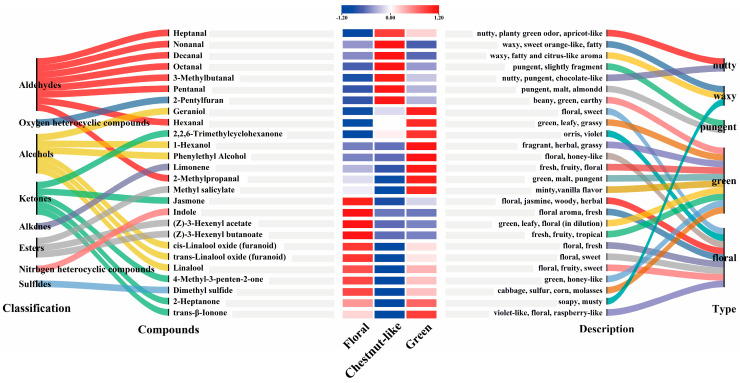
Summary of key aroma-active compounds in HJGT, their odor descriptors, and associated aroma types.

By consulting published literature and reference books on the aroma characteristics and odor thresholds in water of volatile compounds, Table 1 summarizes the volatile compounds with rOAV > 1 detected in the three HJGT aroma types by GC-MS. To accurately characterize the aroma features of each type of HJGT, 26 compounds were selected from the 61 volatile compounds using a rOAV > 1, suggesting their potential as key contributors to the aroma characteristics of the corresponding tea samples. These include linalool (rOAV: 759.64–2072.88), trans-β-ionone (rOAV: 748.00–1210.75), dimethyl sulfide (rOAV: 750.57–1207.34), decanal (rOAV: 161.75–441.43), geraniol (rOAV: 57.29–435.84), 3-methylbutanal (rOAV: 73.11–185.80), nonanal (rOAV: 47.51–126.93), 2,2,6-trimethylcyclohexanone (rOAV: 25.81–55.70), pentanal (rOAV: 18.75–37.95), hexanal (rOAV: 11.62–32.66), and octanal (rOAV: 17.10–30.05). These 11 compounds showed mean rOAV values exceeding 10 in all three aroma types (Table 1), suggesting that they are likely among the major odor-active constituents of HJGT. These 26 aroma compounds comprise eight aldehydes, six alcohols, five ketones, three esters, as well as one alkene, one nitrogen-heterocyclic compound, one oxygen-heterocyclic compound and one sulfur-containing compound. Among them, linalool, trans-linalool oxide (furanoid), cis-linalool oxide (furanoid), indole, and (Z)-jasmone are with distinct floral notes (Figure 6), and they exhibited the highest rOAV values in floral HJGT (Table 1), suggesting they are likely key volatile contributors to its pronounced floral aroma. Interestingly, studies have demonstrated that the relative contents of linalool, trans-linalool oxide (furanoid), and cis-linalool oxide (furanoid) show a strong negative correlation with the fixation temperature during green tea processing [13], specifically, their semi-quantitative values decrease as the fixation temperature increases. In other words, relatively lower fixation temperatures may favour the development of floral qualities in green tea. Additionally, 4-methyl-3-penten-2-one (described as green, honey-like), dimethyl sulfide (cabbage, corn-like), (Z)-3-hexenyl acetate (green), and (Z)-3-hexenyl butanoate (fresh, leafy) also displayed relatively high rOAV values in floral HJGT samples. Together with the floral compounds mentioned above, they likely interact and harmonise to shape the overall floral aroma profile of HJGT. This observation may be partly attributed to the fact that the floral HJGT samples in this study were not further sub-classified by specific floral nuances. During the initial screening, any sample exhibiting a detectable degree of floral character was grouped into the floral category. It should be noted, however, that the predominant odorants may differ among various floral aroma green tea subtypes [22,36,37].

A similar pattern was observed in green HJGT. In contrast to previous studies [13,22], floral-scented compounds such as trans-β-ionone, geraniol, and phenylethyl alcohol did not exhibit the highest rOAV values in the floral tea group. Instead, together with hexanal (green, leafy), 2,2,6-trimethylcyclohexanone (orris), 1-hexanol (fragrant, herbal), limonene (fresh), 2-methylpropanal (green, malt), methyl salicylate (minty), and 2-heptanone (soapy, musty), they showed the highest rOAV values in green tea samples. It is worth noting that although trans-β-ionone is present at low semi-quantitative values, its extremely low OT makes it indispensable for tea aroma formation [38,39]. Although geraniol and linalool originate from the same biosynthetic precursors and possess similar chemical structures and floral odor characteristics, their semi-quantitative profiles differed markedly among the HJGT samples. Previous research has shown that linalool can be oxidised to form linalool oxides (furanoid) or be thermally degraded to yield geraniol [40], while geraniol and phenylethyl alcohol decrease sharply with increasing temperature within a certain range. This indicates that changes in the levels of these compounds are linked to alterations in key non-enzymatic transformation pathways under corresponding processing conditions, particularly those related to temperature [13]. Through complex processing, these compounds interact and contribute in specific proportions, collectively shaping the green HJGT. The reason these samples were initially classified into the green group may be that their green-scented attributes outweighed other aromatic characteristics. This also explains why the rOAV values of potential aroma-active compounds were relatively high in both the floral and green HJGT samples (Figure 6).

In contrast, the potential aroma-active compounds in chestnut-like HJGT exhibited a more distinct profile. Seven volatile compounds showed the highest rOAV values in this group, namely decanal (rOAV: 161.75–441.43), 3-methylbutanal (rOAV: 73.11–185.80), nonanal (rOAV: 47.51–126.93), pentanal (rOAV: 18.75–37.95), octanal (rOAV: 17.10–30.05), heptanal (rOAV: 6.23–26.64), and 2-pentylfuran (rOAV: 2.19–5.88). Among these, six are aldehydes and one is an oxygen-heterocyclic compound. With the exception of 2-pentylfuran (described as beany), the remaining compounds are characterized as waxy, nutty, fruity, or pungent. These compounds are considered key aroma-contributing substances in chestnut-like green tea, which aligns well with earlier findings [41]. However, previous studies have reported a larger number of key aroma compounds, possibly due to differences in the materials, cultivars, and processing methods employed, which could lead to greater variation. In addition, because odor thresholds are not yet available for certain volatiles, future efforts should focus on determining these values to enable a more comprehensive rOAV-based evaluation of their sensory contributions to tea aroma.

**Table 1:** rOAVs of candidate differential volatiles (VIP > 1) identified by PLS-DA in the three HJGT aroma types.

NO.	Volatile Compound	RI-Nist	RI-Test	Category	OT ^#^ (μg/L)	rOAV		
						Floral	Chestnut-like	Green
1	Dodecane	1200	1200	Alkanes	10,000 [42]	<1	<1	<1
2	Tetradecane	1400	1400	Alkanes	1000 [43]	<1	<1	<1
3	Limonene	1030	1030	Alkenes	10 [25]	1.61	1.23	2.70
4	(E)-4,8-Dimethylnona-1,3,7-triene	1116	1118	Alkenes	n.f.	-	-	-
5	β-Elemene	1391	1395	Alkenes	n.f.	-	-	-
6	Caryophyllene	1419	1423	Alkenes	64 [44]	<1	<1	<1
7	Humulene	1454	1458	Alkenes	390 [45]	<1	<1	<1
8	α-Farnesene	1508	1511	Alkenes	87 [46]	<1	<1	<1
9	α-Corocalene	1623	1633	Alkenes	n.f.	-	-	-
10	2-Methyl-3-pentanol	775	767	Alcohols	420 [47]	<1	<1	<1
11	(Z)-3-Hexenol	857	853	Alcohols	n.f.	-	-	-
12	1-Hexanol	868	866	Alcohols	5.6 [48]	2.65	2.54	5.51
13	cis-Anhydrolinalool oxide	1008	992	Alcohols	n.f.	-	-	-
14	2-Ethylhexanol	1030	1030	Alcohols	n.f.	-	-	-
15	Octanol	1071	1073	Alcohols	n.f.	-	-	-
16	cis-Linalool oxide (furanoid)	1074	1073	Alcohols	6 [49]	3.85	<1	2.61
17	trans-Linalool oxide (furanoid)	1086	1089	Alcohols	6 [49]	10.29	2.03	6.87
18	Linalool	1099	1101	Alcohols	0.22 [48]	2072.88	759.64	1753.28
19	Phenylethyl alcohol	1116	1114	Alcohols	60 [50]	<1	<1	1.69
20	cis-Linalol oxide (pyranoid)	1174	1175	Alcohols	n.f.	-	-	-
21	trans-Linalol oxide (pyranoid)	1173	1175	Alcohols	3000 [51]	<1	<1	<1
22	Geraniol	1255	1256	Alcohols	1.1 [52]	57.29	203.94	435.84
23	2-Methylpropanal		583	Aldehydes	0.49 [52,53]	8.67	6.36	12.19
24	3-Methylbutanal	652	652	Aldehydes	0.2 [50]	73.11	185.80	109.46
25	Pentanal	699	701	Aldehydes	2.7 [7]	18.75	37.95	23.59
26	2-Methyl-2-butenal	746	740	Aldehydes	500 [54]	<1	<1	<1
27	Hexanal	800	802	Aldehydes	4.5 [25,48]	11.62	22.16	32.66
28	2-Methyl-2-pentenal	837	830	Aldehydes	290 [55]	<1	<1	<1
29	Heptanal	901	902	Aldehydes	2.8 [48]	6.23	26.64	19.44
30	Octanal	1003	1004	Aldehydes	0.32 [7]	17.10	30.05	18.98
31	Nonanal	1104	1105	Aldehydes	0.32 [7]	58.18	126.93	47.51
32	Decanal	1206	1207	Aldehydes	0.1 [56]	184.85	441.43	161.75
33	Geranial	1276	1272	Aldehydes	32 [11]	<1	<1	<1
34	2-Methyl-3-pentanone	745	747	Ketones	40 [55]	<1	<1	<1
35	4-Methyl-3-penten-2-one	798	801	Ketones	200 [51]	1.19	<1	<1
36	2-Heptanone	891	891	Ketones	1 [51]	6.34	2.14	6.92
37	6-Methyl-5-heptene-2-one	986	987	Ketones	50 [49,50]	<1	<1	<1
38	2,2,6-Trimethylcyclohexanone	1036	1035	Ketones	0.1 [57]	25.81	42.44	55.70
39	(3E)-5-Ethyl-6-methyl-3-hepten-2-one	1144	1148	Ketones	n.f.	-	-	-
40	(Z)-Jasmone	1394	1401	Ketones	1.9 [58]	14.52	3.33	7.44
41	Geranyl acetone	1453	1455	Ketones	60 [59]	<1	<1	<1
42	trans-β-Ionone	1491	1489	Ketones	0.007 [54]	1041.21	748.00	1210.75
43	Butyl ethanoate	812	813	Esters	58 [60]	<1	<1	<1
44	Methyl hexanoate	925	924	Esters	70 [44]	<1	<1	<1
45	(Z)-3-Hexenyl acetate	1005	1009	Esters	13 [52]	19.36	<1	1.81
46	(Z)-3-Hexenyl butanoate	1187	1188	Esters	50 [61]	9.90	<1	<1
47	Hexyl butanoate	1192	1193	Esters	203 [44]	<1	<1	<1
48	Methyl salicylate	1192	1195	Esters	40 [49,62]	1.90	<1	3.92
49	(3Z)-3-Hexenyl 2-methylbutanoate	1234	1234	Esters	n.f.	-	-	-
50	(Z)-3-Hexenyl pentanoate	1237	1238	Esters	n.f.	-	-	-
51	2-Phenylethyl acetate	1258	1259	Esters	249.59 [60]	<1	<1	<1
52	(Z)-3-Hexenyl hexanoate	1380	1383	Esters	n.f.	-	-	-
53	Cyclohexyl hexanoate	1405	1387	Esters	n.f.	-	-	-
54	(E)-2-Hexenyl hexanoate	1391	1390	Esters	781 [63]	<1	<1	<1
55	(Z)-3-Hexenyl benzoate	1570	1578	Esters	n.f.	-	-	-
56	2-Ethylfuran	703	704	Oxygen heterocyclic compounds	8000 [48]	<1	<1	<1
57	2,3-Dimethylfuran		716	Oxygen heterocyclic compounds	n.f.	-	-	-
58	2-Pentylfuran	993	991	Oxygen heterocyclic compounds	4.8 [64]	2.19	5.88	3.12
59	2-Ethyl-4-methyl-1H-pyrrole	938	925	Nitrogen heterocyclic compounds	n.f.	-	-	-
60	Indole	1295	1295	Nitrogen heterocyclic compounds	11 [51]	8.78	<1	<1
61	Dimethyl sulfide		569	Sulfides	1.1 [50]	1207.34	750.57	1071.84

^#^ Odor thresholds were obtained from previously reported sources. (n.f., not found in the available literature; -, rOAV not calculated due to absence of threshold data).

### 3.5. Characteristic Volatiles Associated with Each Aroma Type

**Figure 7:**
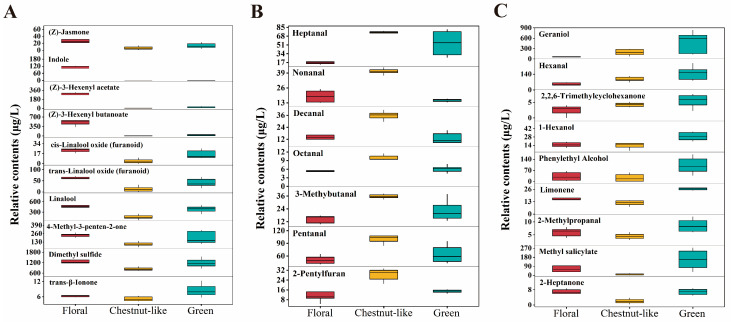
Boxplots of key volatile compounds in floral (**A**), chestnut-like (**B**) and green (**C**) HJGT samples.

To ensure statistical rigor, one-way analysis of variance (ANOVA) with the F-test [65] was applied to further examine compounds with VIP > 1 and rOAV > 1 (Table S3). As shown in Figure 7, all 26 previously screened compounds showed *p*-values below 0.05, confirming statistically significant differences among the three groups of HJGT samples. These were therefore identified as marker volatile compounds differentiating for the aroma types.

The key contributing volatiles in HJGT samples are presented in Figure 7. Ten major volatile components were identified for the floral type, including (Z)-jasmone, cis-linalool oxide (furanoid), trans-linalool oxide (furanoid), linalool, 4-methyl-3-penten-2-one, trans-β-ionone, (Z)-3-hexenyl acetate, (Z)-3-hexenyl butanoate, indole, and dimethyl sulfide. Except for trans-β-ionone, the other nine volatiles exhibited the highest semi-quantitative values in the floral HJGT samples. Previous studies have confirmed that most of these compounds, such as (Z)-jasmone, cis-linalool oxide (furanoid), trans-linalool oxide (furanoid), linalool, trans-β-ionone, (Z)-3-hexenyl acetate, and indole, possess distinct floral scent characteristics. Notably, trans-β-ionone arises from the heat-induced breakdown of β-carotene during tea manufacture [5,40]. Characterized by a distinct violet-like floral note, trans-β-ionone in the present study was found at relatively high levels in both the green and floral tea groups. As previously reported, this compound is a key volatile responsible for the floral aroma in green tea [22]. Therefore, trans-β-ionone was considered one of the key aroma-contributing compounds in floral HJGT samples in this work.

Seven major volatiles were identified in the chestnut-like HJGT samples, including heptanal, nonanal, decanal, octanal, 3-methylbutanal, pentanal, and 2-pentylfuran. These compounds exhibited the highest semi-quantitative value in chestnut-like HJGT samples. Pentanal has been previously reported as an important contributor to the cooked corn-like aroma of green tea [66]. In the present study, pentanal was also recognized as a key volatile in chestnut-like HJGT samples, marking its first identification as a critical aroma-active substance in this particular green tea aroma type. The other six volatiles have been frequently reported in chestnut-like green teas.

Nine of the 26 compounds were identified as characteristic of the green teas, namely geraniol, hexanal, limonene, 1-hexanol, methyl salicylate, phenylethyl alcohol, 2-heptanone, 2-methylpropanal, and 2,2,6-trimethylcyclohexanone. These volatiles reached their highest levels in green HJGT samples, suggesting that they contribute considerably to the fresh-like odor quality of this tea type. Their aroma profiles are mainly described as fragrant, minty, fresh, and floral. Compared with the floral and chestnut-like tea samples, the green teas did not exhibit a single dominant aromatic trait but rather presented a subtle, blended aromatic impression, these findings are highly consistent with previous aroma studies that also utilized HJGT as the experimental material [67]. Research has shown that compounds with similar chemical structures or odor characteristics can produce synergistic or additive effects, thereby enhancing the intensity of a particular scent [68]. It is therefore plausible that hexanal, 2-methylpropanal, limonene, and methyl salicylate, all associated with fresh odor notes may act synergistically or additively. Similarly, geraniol, 1-hexanol, phenylethyl alcohol, 2-heptanone, and 2,2,6-trimethylcyclohexanone imparting aromatic qualities, could also interact in a synergistic or additive manner. However, exactly how these aroma constituents collectively construct the green-scented quality of HJGT requires further investigation, integrating analyses of concentration, molecular structure, and the response profiles of olfactory receptor neurons [69,70].

These observations illustrate that the rOAV method has certain limitations when used alone to characterize flavor compounds in samples. Combining it with GC-O would allow more accurate identification of the characteristic flavor components [50,71]. Furthermore, subsequent studies should include validation experiments and systematically collect more tea samples with typical fresh aroma profiles, comparing and verifying the present findings through aroma recombination and omission tests.

## 4. Conclusions

In this study, HS-SPME-GC-MS was employed for comprehensive volatile profiling of three HJGT aroma types. A total of 111 common volatiles, belonging to 10 chemical classes, were identified across 26 representative tea samples. The volatile profiles of the three aroma types were clearly distinguishable. A PLS-DA model constructed on the basis of volatile composition effectively separated the three groups. Combined with rOAV analysis, 26 compounds with VIP > 1 and *p* < 0.05 were identified as key differential aroma components.

Among these, volatiles including (Z)-jasmone, cis-linalool oxide (furanoid), trans-linalool oxide (furanoid), linalool, 4-methyl-3-penten-2-one, trans-β-ionone, (Z)-3-hexenyl acetate, (Z)-3-hexenyl butanoate, indole, and dimethyl sulfide were enriched in the floral teas, contributing to their floral aroma. Compounds such as heptanal, nonanal, decanal, octanal, 3-methylbutanal, pentanal, and 2-pentylfuran were enriched in the chestnut-like teas, forming their characteristic chestnut-like aroma. Meanwhile, geraniol, hexanal, limonene, 1-hexanol, methyl salicylate, phenylethyl alcohol, 2-heptanone, 2-methylpropanal and 2,2,6-trimethylcyclohexanone were enriched in the green aroma teas, contributing to their green aroma profile. These compounds thus serve as promising volatile markers for discriminating HJGT types.

Nevertheless, the present identification of candidate aroma-active compounds is based on GC-MS quantification and rOAV calculations without direct sensory validation through GC-O, aroma recombination, or omission experiments. In addition, the sample set was limited to commercially sourced teas with relatively small and uneven group sizes, and the quantification of volatiles was performed using a single-point internal standard method, yielding semi-quantitative estimates rather than absolute concentrations. These considerations should be taken into account when interpreting the results. The compounds identified herein should therefore be viewed as statistically supported candidates deserving of further verification, rather than as definitively confirmed aroma contributors.

Despite these limitations, our findings provide a robust screening-level framework for understanding the volatile basis of HJGT aroma differentiation. The study offers a comprehensive dataset and a prioritized list of candidate markers that can guide future mechanistic studies and targeted sensory validation. Future work integrating GC-O analysis, aroma recombination and omission tests with authentic standards, alongside expanded and more balanced sampling across diverse origins and cultivars, will be essential to definitively establish the sensory relevance of these candidate compounds and to fully elucidate the mechanisms underlying HJGT aroma formation.

## Data Availability

The original contributions presented in this study are included in the article/supplementary materials. Further inquiries can be directed to the corresponding authors.

## Supplementary Materials

The following supporting information can be downloaded at: https://www.mdpi.com/article/10.3390/foods15152650/s1, Table S1: The sensory evaluation results of three aroma types in Huangjin green tea samples; Table S2: Information of 111 volatile compounds (μg/L); Table S3: A total of 26 key aroma compounds were identified as candidates, based on the criteria of VIP> 1, rOAV> 1, and *P*< 0.05; Figure S1: TICs of the pooled quality control (QC) samples representing the three aroma types of HJGT. A: QC-1. B:QC-2. C: QC-3; Figure S2: EI mass spectra of 26 key aroma compounds and the corresponding reference spectrum from the NIST20 spectral library. A: EI mass spectrum of the compounds in the sample. B: EI mass spectrum of the compounds matched against the NIST20 spectral library.
