# Supplementary material for: Unraveling the Diversity of Characteristic Aroma Components in Huangjin Green Tea Across Different Flavor Categories
Source: Foods. 2026 Jul 28;15(15):2650. doi: 10.3390/foods15152650 (PMC13464752; doi:10.3390/foods15152650)
Supplement: Supplementary file 1 [file foods-15-02650-s001.zip › foods-4409664-supplementary.pdf]

Unraveling the Diversity of Characteristic Aroma Components in Huangjin Green Tea Across Different Flavor Categories

Table S1. The sensory evaluation results of three aroma types in Huangjin green tea samples.

No.	Samples		Aroma Description	Aroma Intensity	Score	No.	Samples		Aroma Description	Aroma Intensity	Score
1	Flora	F1	Rosy flavor	Strong	95.0	14	Chestnut-like	C8	Chestnut-like flavor	Strong	93.0
2		F2	Honeysuckle-like flavor	Moderate	91.0	15		C9	Tender Chestnut-like flavor	Strong	94.0
3		F3	Orchid-like flavor	Strong	94.0	16		C10	Chestnut-like flavor	Strong	93.0
4		F4	Orchid-like flavor	Strong	93.0	17		C11	Chestnut-like flavor	Strong	94.0
5		F5	Flowery flavor	Strong	93.0	18		C12	Chestnut-like flavor	Strong	93.0
6		F6	Flowery flavor	Strong	93.0	19	Green	G1	Delicate Fragrance	Strong	93.0
7	Chestnut-like	C1	Tender Chestnut-like flavor	Strong	94.0	20		G2	Delicate Fragrance	Strong	94.0
8		C2	Chestnut-like flavor	Moderate	91.0	21		G3	Delicate and lasting high aroma	Strong	95.0
9		C3	Chestnut-like flavor	Moderate	91.0	22		G4	Delicate Fragrance	Strong	93.0
10		C4	Chestnut-like flavor	Moderate	91.0	23		G5	Delicate and refined fragrance	Strong	94.0
11		C5	Chestnut-like flavor	Strong	92.0	24		G6	Delicate Fragrance	Moderate	91.0
12		C6	Tender Chestnut-like Aroma	Strong	94.0	25		G7	Delicate Fragrance	Strong	95.0
13		C7	Tender Chestnut-like Aroma	Strong	94.0	26		G8	Delicate Fragrance	Strong	95.0

Table S2. Information of 111 volatile compounds (μg/L)

NO.	Name	CAS	RT	RI-Nist	RI-test	Category	Floral	Chestnut-like	Green	VIP
1	2-Cyanobutane	18936-17-9	4.7896	723	722	Alkanes	0.78±0.14	0.42±0.19	0.58±0.24	0.951
2	4-Methyloctane	2216-34-4	8.9079	863	860	Alkanes	2.15±1.29	3.00±3.61	2.77±3.46	0.197
3	Decane	124-18-5	16.4087	1000	1000	Alkanes	9.72±8.10	6.64±9.25	24.08±13.05	0.991
4	5-Ethyldecane	17302-36-2	24.9724	1139	1146	Alkanes	0.36±0.24	0.26±0.29	0.90±0.48	0.951
5	Dodecane	112-40-3	27.9159	1200	1200	Alkanes	9.67±6.37	7.52±6.73	32.83±16.49	1.165
6	3-Methyltridecane	6418-41-3	35.9718	1371	1371	Alkanes	0.57±0.37	0.42±0.32	2.24±1.74	0.999
7	Tetradecane	629-59-4	37.2542	1400	1400	Alkanes	5.88±2.41	3.49±1.98	16.50±9.80	1.162
8	Pentadecane	629-62-9	41.4069	1500	1500	Alkanes	2.83±0.78	2.17±0.63	3.07±0.94	0.772
9	2,4-Dimethyl-1-heptene	19549-87-2	8.0257	836	837	Alkenes	2.40±1.39	4.61±5.42	2.62±1.62	0.480
10	2-Pinene	80-56-8	12.2947	937	930	Alkenes	3.18±1.01	1.74±0.65	3.16±2.03	0.912
11	Myrcene	123-35-3	15.8665	991	991	Alkenes	40.92±10.33	49.07±26.38	80.27±42.38	0.814
12	p-Cymene	99-87-6	17.9407	1025	1026	Alkenes	9.24±2.40	9.84±3.12	12.53±4.21	0.750
13	Limonene	138-86-3	18.1989	1030	1030	Alkenes	16.10±4.05	12.29±5.41	21.12±7.50	1.042
14	(E)-β-Ocimene	3779-61-1	18.8315	1049	1040	Alkenes	9.75±3.02	9.77±5.03	16.77±8.89	0.811
15	β-Ocimene	13877-91-3	19.4512	1037	1050	Alkenes	42.77±17.34	23.18±10.99	32.52±14.08	0.886
16	(E)-4,8-Dimethylnona-1,3,7-triene	19945-61-0	23.4533	1116	1118	Alkenes	33.18±16.17	8.97±7.55	5.22±3.08	1.126
17	2,6-Dimethyl-2,4,6-octatriene	673-84-7	24.8260	1131	1131	Alkenes	4.22±1.49	3.06±1.42	4.57±2.55	0.712
18	α-Cubebene	17699-14-8	35.1283	1351	1352	Alkenes	1.17±0.37	4.88±1.65	9.53±5.80	0.992
19	Copaene	3856-25-5	36.3117	1376	1379	Alkenes	0.96±0.33	2.02±1.38	4.02±3.46	0.805
20	β-Elemene	515-13-9	37.0175	1391	1395	Alkenes	2.57±1.20	0.18±0.06	0.35±0.28	1.189
21	Caryophyllene	87-44-5	38.2224	1419	1423	Alkenes	1.66±1.06	1.48±0.71	3.16±1.40	1.004
22	Humulene	6753-98-6	39.6511	1454	1458	Alkenes	17.8±9.26	2.16±0.75	3.11±2.01	1.207
23	α-Muurolene	10208-80-7	41.5446	1499	1504	Alkenes	6.32±2.21	5.71±1.67	5.61±1.88	0.414

NO.	Name	CAS	RT	RI-Nist	RI-test	Category	Floral	Chestnut-like	Green	VIP
24	α-Farnesene	502-61-4	41.8028	1508	1511	Alkenes	1.31±0.50	0.34±0.19	0.63±0.30	1.230
25	Butylated Hydroxytoluene	128-37-0	42.0223	1513	1516	Alkenes	12.35±10.83	4.40±2.01	6.73±2.42	0.962
26	δ-Cadinene	483-76-1	42.4784	1524	1529	Alkenes	6.55±3.34	41.53±14.23	64.72±47.46	0.927
27	Cubenene	29837-12-5	42.8356	1532	1539	Alkenes	0.46±0.15	3.13±1.67	6.96±5.62	0.902
28	α-Calacorene	21391-99-1	43.2702	1542	1550	Alkenes	0.72±0.36	3.50±1.66	6.65±5.65	0.826
29	α-Corocalene	20129-39-9	45.7705	1623	1633	Alkenes	0.15±0.06	0.51±0.22	1.04±0.63	1.099
30	Cadalene	483-78-3	46.8291	1674	1685	Alkenes	0.39±0.28	1.13±0.45	1.69±1.04	0.910
31	Toluene	108-88-3	5.7536	763	765	Aromatic hydrocarbons	47.18±43.62	10.67±4.26	14.45±4.35	0.961
32	Ethylbenzene	100-41-4	8.8778	855	859	Aromatic hydrocarbons	2.59±1.25	2.02±1.06	3.51±2.31	0.629
33	1,4-Dimethyl benzene	106-42-3	9.2092	865	867	Aromatic hydrocarbons	7.06±2.65	4.53±2.58	7.69±3.23	0.796
34	1,2-Dimethylbenzene	95-47-6	10.2678	887	894	Aromatic hydrocarbons	1.73±0.64	1.41±0.76	2.38±0.84	0.902
35	1-Ethyl-2,3-dimethylbenzene	933-98-2	21.5942	1094	1086	Aromatic hydrocarbons	10.01±10.27	1.15±0.45	13.55±10.38	0.699
36	Naphthalene	91-20-3	26.9734	1182	1183	Aromatic hydrocarbons	12.82±8.23	5.97±7.60	13.70±12.38	0.729
37	1-Pentanol	71-41-0	5.6761	765	761	Alcohols	64.79±18.15	68.94±15.76	93.12±19.49	0.896
38	2-Methyl-3-pentanol	565-67-3	5.8009	775	767	Alcohols	0.64±0.17	1.24±0.64	2.52±0.73	1.129
39	(Z)-3-Hexenol	928-96-1	8.6024	857	853	Alcohols	238.37±99.05	16.51±13.45	97.83±62.97	1.273
40	1-Hexanol	111-27-3	9.1403	868	866	Alcohols	14.84±4.28	14.63±5.98	30.86±10.16	1.108
41	1-Octen-3-ol	3391-86-4	15.1005	980	978	Alcohols	31.72±14.4	40.57±30.49	72.76±47.04	0.696
42	cis-Anhydrolinalool oxide	54750-69-5	15.9482	1008	992	Alcohols	1.68±0.44	0.68±0.20	1.05±0.47	1.134
43	2-Ethylhexanol	104-76-7	18.2677	1030	1030	Alcohols	10.09±7.52	24.55±7.75	11.16±5.93	1.135
44	Phenylacetaldehyde	122-78-1	19.1714	1405	1046	Alcohols	7.88±2.53	11.09±7.64	17.41±6.44	0.809
45	Octanol	111-87-5	20.8024	1071	1073	Alcohols	7.65±4.68	7.32±2.93	14.85±4.42	1.079
46	cis-Linalool oxide (furanoid)	5989-33-3	20.8799	1074	1073	Alcohols	23.10±4.06	4.94±3.05	15.69±6.45	1.355

NO.	Name	CAS	RT	RI-Nist	RI-test	Category	Floral	Chestnut-like	Green	VIP
47	trans-Linalool oxide (furanoid)	34995-77-2	21.8008	1086	1089	Alcohols	61.75±11.65	13.27±9.74	41.21±17.52	1.324
48	Linalool	78-70-6	22.4850	1099	1101	Alcohols	456.03±56.58	167.12±74.21	385.72±101.27	1.354
49	Phenylethyl Alcohol	60-12-8	23.2295	1116	1114	Alcohols	37.46±24.31	36.11±30.77	105.74±46.13	1.143
50	cis-Linalol oxide (pyranoid)	14009-71-3	26.2935	1174	1175	Alcohols	1.33±0.58	0.31±0.14	0.59±0.26	1.117
51	trans-Linalol oxide (pyranoid)	39028-58-5	26.5603	1173	1175	Alcohols	12.35±4.91	2.48±1.51	5.39±2.26	1.221
52	Terpineol	98-55-5	27.4296	1189	1191	Alcohols	13.25±4.49	12.08±8.81	20.67±15.39	0.747
53	Nerol	106-25-2	29.3618	1228	1230	Alcohols	5.08±2.37	10.99±8.88	23.72±17.97	0.828
54	Geraniol	106-24-1	30.6442	1255	1256	Alcohols	63.02±28.30	224.34±157.43	441.92±330.42	1.078
55	2,4-Di-tert-butylphenol	96-76-4	41.9362	1519	1514	Alcohols	5.40±3.31	4.24±2.24	7.77±3.62	0.753
56	Nerolidol	40716-66-3	43.9846	1564	1570	Alcohols	4.87±2.35	3.61±3.20	1.28±0.96	0.918
57	Cedrol	77-53-2	45.2928	1598	1609	Alcohols	1.87±0.89	1.23±0.56	1.34±1.05	0.621
58	α-epi-Muurolol	19912-62-0	46.1922	1642	1654	Alcohols	0.21±0.11	0.30±0.12	0.40±0.24	0.729
59	2-Methylpropanal	78-84-2	2.9951		583	Aldehydes	6.07±1.65	4.45±1.70	8.53±2.50	1.110
60	3-Methylbutanal	590-86-3	3.7439	652	652	Aldehydes	14.62±4.21	39.72±9.21	21.89±9.63	1.222
61	2-Methylbutanal	96-17-3	3.8558	662	661	Aldehydes	41.45±7.88	40.1±17.25	59.87±20.15	0.762
62	Pentanal	110-62-3	4.2990	699	701	Aldehydes	50.63±16.66	102.48±19.94	66.47±22.93	1.218
63	2-Methyl-2-butenal	1115-11-3	5.2027	746	740	Aldehydes	2.29±0.61	1.44±0.79	4.27±1.30	1.238
64	2-Methylpental	123-15-9	5.5728	760	758	Aldehydes	5.55±1.69	3.58±2.56	4.39±3.46	0.647
65	Hexanal	66-25-1	6.6013	800	802	Aldehydes	52.29±11.52	99.74±43.1	166.96±53.85	1.165
66	2-Methyl-2-pentenal	623-36-9	7.7417	837	830	Aldehydes	0.95±0.47	2.21±1.65	7.15±3.94	1.048
67	Heptanal	111-71-7	10.6336	901	902	Aldehydes	18.47±5.49	73.76±24.08	54.42±23.31	1.034
68	Octanal	124-13-0	16.6325	1003	1004	Aldehydes	5.47±1.69	9.62±3.68	4.47±1.43	1.017
69	2,6-Dimethyl-5-heptenal	106-72-9	19.7868	1052	1055	Aldehydes	1.75±1.42	15.85±8.83	12.15±8.67	0.828
70	Nonanal	124-19-6	22.7475	1104	1105	Aldehydes	18.62±5.85	33.95±12.84	18.67±4.71	1.002
71	Decanal	112-31-2	28.2214	1206	1207	Aldehydes	14.79±4.77	27.81±8.60	12.94±5.87	1.190

NO.	Name	CAS	RT	RI-Nist	RI-test	Category	Floral	Chestnut-like	Green	VIP
72	β-Cyclocitral	432-25-7	28.9659	1220	1222	Aldehydes	1.94±1.35	4.26±2.41	4.98±3.06	0.644
73	Geranial	141-27-5	31.4618	1276	1272	Aldehydes	1.73±0.70	5.89±3.44	13.88±6.43	1.123
74	2-Pentanone	107-87-9	4.1785	685	690	Ketones	1.01±0.35	0.69±0.40	1.19±0.71	0.758
75	2,3-Pentanedione	600-14-6	4.2646	698	700	Ketones	1.77±0.47	1.68±0.57	1.81±0.62	0.571
76	2-Methyl-3-pentanone	565-69-5	5.3490	745	747	Ketones	34.97±6.25	13.79±5.03	29.87±12.36	1.267
77	4-Methyl-3-penten-2-one	141-79-7	6.5884	798	801	Ketones	237.00±29.58	95.56±28.51	195.02±90.70	1.238
78	2-Heptanone	110-43-0	10.1473	891	891	Ketones	6.34±2.27	2.57±1.40	6.92±1.58	1.252
79	1-Octen-3-one	4312-99-6	14.7648	979	972	Ketones	7.46±5.05	15.69±13.49	18.55±13.91	0.660
80	2,3-Octanedione	585-25-1	15.4490	984	983	Ketones	12.07±5.21	9.99±3.88	13.02±4.20	0.630
81	6-Methyl-5-heptene-2-one	110-93-0	15.6556	986	987	Ketones	7.03±2.95	6.00±2.40	12.68±3.47	1.135
82	2,2,6-Trimethylcyclohexanone	2408-37-9	18.5604	1036	1035	Ketones	3.32±0.64	4.24±0.74	6.37±1.62	1.233
83	(3E)-5-Ethyl-6-methyl-3-hepten-2-one	57283-79-1	25.1015	1144	1148	Ketones	0.72±0.17	0.87±0.49	2.34±0.66	1.269
84	(Z)-Jasmone	488-10-8	37.3101	1394	1401	Ketones	27.59±16.03	6.51±5.59	14.14±10.26	1.023
85	Geranyl acetone	3796-70-1	39.5478	1453	1455	Ketones	19.86±15.92	4.86±1.60	14.83±8.89	1.060
86	trans-β-Ionone	79-77-6	40.9550	1491	1489	Ketones	7.72±2.73	5.24±1.26	8.48±2.64	1.091
87	6,10,14-Trimethyl-2-pentadecanone	502-69-2	48.9980	1844	1850	Ketones	2.89±1.36	1.73±1.04	2.52±1.72	0.600
88	Butyl ethanoate	123-86-4	7.0876	812	813	Esters	35.85±27.41	2.74±2.05	3.73±1.79	1.003
89	Methyl hexanoate	106-70-7	11.8385	925	924	Esters	3.70±1.54	0.20±0.11	1.00±0.42	1.250
90	(Z)-3-Hexenyl acetate	3681-71-8	16.9423	1005	1009	Esters	251.67±109.58	12.50±9.14	23.53±12.60	1.243
91	(Z)-3-Hexenyl butanoate	16491-36-4	27.2661	1187	1188	Esters	495.00±109.51	5.47±3.60	28.24±21.32	1.401
92	Hexyl butanoate	2639-63-6	27.5673	1192	1193	Esters	5.51±1.96	0.19±0.13	1.50±0.94	1.297
93	Methyl salicylate	119-36-8	27.6534	1192	1195	Esters	76.02±57.77	12.87±5.53	131.02±114.66	1.032
94	(3Z)-3-Hexenyl 2-methylbutanoate	53398-85-9	29.5813	1234	1234	Esters	11.50±2.40	3.14±1.54	13.46±7.55	1.269
95	(Z)-3-Hexenyl pentanoate	35852-46-1	29.7577	1237	1238	Esters	13.70±3.48	0.48±0.14	1.50±1.21	1.347
96	2-Phenylethyl acetate	103-45-7	30.7905	1258	1259	Esters	8.89±5.54	2.21±1.36	3.61±1.97	1.066

NO.	Name	CAS	RT	RI-Nist	RI-test	Category	Floral	Chestnut-like	Green	VIP
97	Methyl (E)-geraniate	1189-09-9	33.9363	1324	1326	Esters	4.01±1.22	3.39±1.98	3.43±2.20	0.520
98	(Z)-3-Hexenyl hexanoate	31501-11-8	36.4882	1380	1383	Esters	1181.74±383.93	15.27±6.70	58.68±32.52	1.383
99	Cyclohexyl hexanoate	6243-10-3	36.6947	1405	1387	Esters	17.30±6.32	0.81±0.60	1.67±0.72	1.247
100	(E)-2-Hexenyl hexanoate	53398-86-0	36.8496	1391	1390	Esters	42.93±15.24	1.15±0.91	8.20±6.85	1.279
101	(Z)-3-Hexenyl benzoate	25152-85-6	44.2858	1570	1578	Esters	7.36±1.71	0.47±0.21	0.86±0.53	1.300
102	Isobutyl phthalate	84-69-5	49.2863	1870	1878	Esters	7.84±2.75	7.87±1.88	7.92±1.92	0.568
103	2-Methylfuran	534-22-5	3.2791	606	617	Oxygen heterocyclic compounds	2.90±2.31	3.93±3.65	4.93±3.74	0.323
104	2-Ethylfuran	3208-16-0	4.3765	703	704	Oxygen heterocyclic compounds	8.91±1.93	11.4±4.48	24.96±7.84	1.200
105	2,3-Dimethylfuran	14920-89-9	4.6347		716	Oxygen heterocyclic compounds	2.59±0.53	1.12±0.74	1.82±0.96	1.024
106	2-Pentylfuran	3777-69-3	15.8880	993	991	Oxygen heterocyclic compounds	11.15±3.02	27.06±7.70	12.24±5.32	1.188
107	1-Ethylpyrrole	617-92-5	7.1220	821	815	Nitrogen heterocyclic compounds	20.24±5.71	14.56±8.05	27.82±11.62	0.924
108	2-Ethyl-4-methyl-1H-pyrrole	69687-77-0	11.9547	938	925	Nitrogen heterocyclic compounds	1.88±0.42	0.94±0.38	1.75±0.64	1.100
109	Indole	120-72-9	32.5205	1295	1295	Nitrogen heterocyclic compounds	115.86±26.60	2.64±3.27	2.18±2.24	1.086
110	Dimethyl sulfide	75-18-3	2.8359		569	Sulphides	1328.07±246.64	825.63±180.92	1179.03±269.77	1.120
111	Dimethyl disulfide	624-92-0	5.2501	746	743	Sulphides	0.63±0.20	1.22±0.54	1.18±0.54	0.693

Table S3. A total of 26 key aroma compounds were identified as candidates, based on the criteria of VIP> 1, rOAV> 1, and $P < 0.05$

NO.	Volatile Compound	Content (μg/L)			*p*-Value
		Floral	Chestnut-like	Green	
1	Limonene	16.10±4.05	12.29±5.41	21.12±7.50	0.000
2	1-Hexanol	14.84±4.28	14.63±5.98	30.86±10.16	0.000
3	cis-Linalool oxide (furanoid)	22.54±4.95	4.94±3.05	15.69±6.45	0.000
4	trans-Linalool oxide (furanoid)	61.75±11.65	13.27±9.74	41.21±17.52	0.000
5	Linalool	456.03±56.58	149.34±88.36	385.72±101.27	0.000
6	Phenylethyl Alcohol	37.46±24.31	36.11±30.77	105.74±46.13	0.000
7	Geraniol	63.02±28.30	224.34±157.43	441.92±330.42	0.000
8	2-Methylpropanal	6.07±1.65	4.45±1.70	8.53±2.50	0.012
9	3-Methylbutanal	14.62±4.21	39.72±9.21	21.89±9.63	0.023
10	Pentanal	50.63±16.66	102.48±19.94	66.47±22.93	0.045
11	Hexanal	52.29±11.52	99.74±43.1	166.96±53.85	0.000
12	Heptanal	18.47±5.49	73.76±24.08	54.42±23.31	0.000
13	Octanal	5.47±1.69	9.62±3.68	4.47±1.43	0.038
14	Nonanal	18.62±5.85	33.95±12.84	18.67±4.71	0.000
15	Decanal	14.79±4.77	27.81±8.60	12.94±5.87	0.000
16	4-Methyl-3-penten-2-one	237.00±29.58	95.56±28.51	195.02±90.70	0.000
17	2-Heptanone	6.34±2.27	2.57±1.40	6.92±1.58	0.001
18	2,2,6-Trimethylcyclohexanone	3.32±0.64	4.24±0.74	6.37±1.62	0.005

19	(Z)-Jasmone	27.59±16.03	6.51±5.59	14.14±10.26	0.000
20	trans-β-Ionone	7.72±2.73	5.24±1.26	8.48±2.64	0.008
21	(Z)-3-Hexenyl acetate	251.67±109.58	12.50±9.14	23.53±12.60	0.000
22	(Z)-3-Hexenyl butanoate	495.00±109.51	5.47±3.60	28.24±21.32	0.000
23	Methyl salicylate	76.02±57.77	12.87±5.53	131.02±114.66	0.000
24	2-Pentylfuran	11.15±3.02	27.06±7.70	12.24±5.32	0.000
25	Indole	115.86±26.60	2.64±3.27	2.18±2.24	0.000
26	Dimethyl sulfide	1328.07±246.64	825.63±180.92	1179.03±269.77	0.000

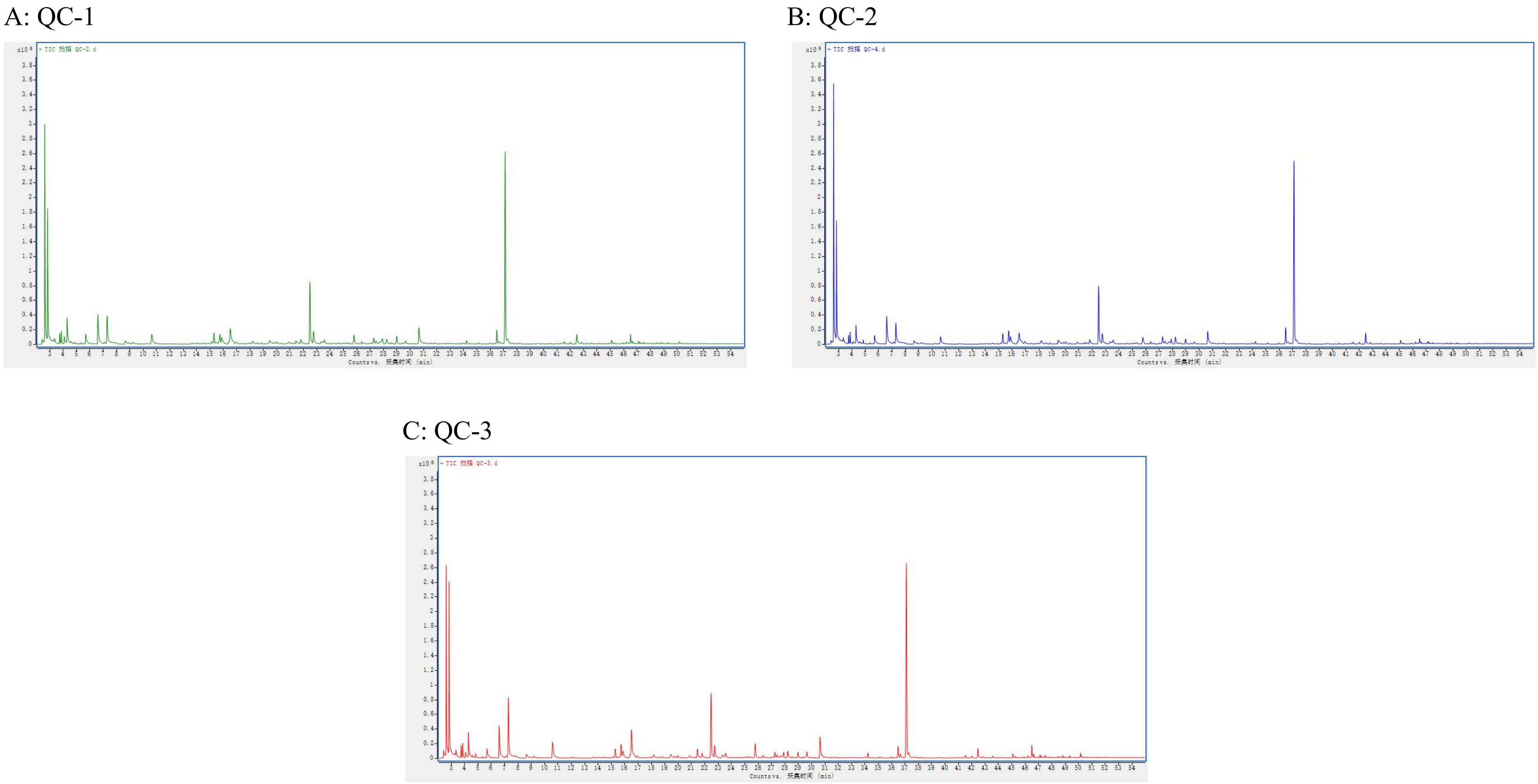

Figure S1. TICs of the pooled quality control (QC) samples representing the three aroma types of HJGT. A: QC-1; B:QC-2; C: QC-3.

(Z)-Jasmone:

A

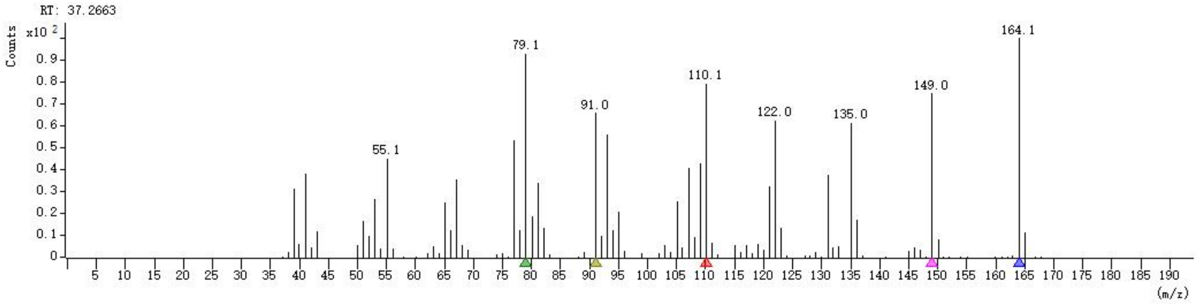
RT: 37.2663
Counts x10 2
79.1
91.0
110.1
122.0
135.0
149.0
164.1
55.1
(m/z)

B

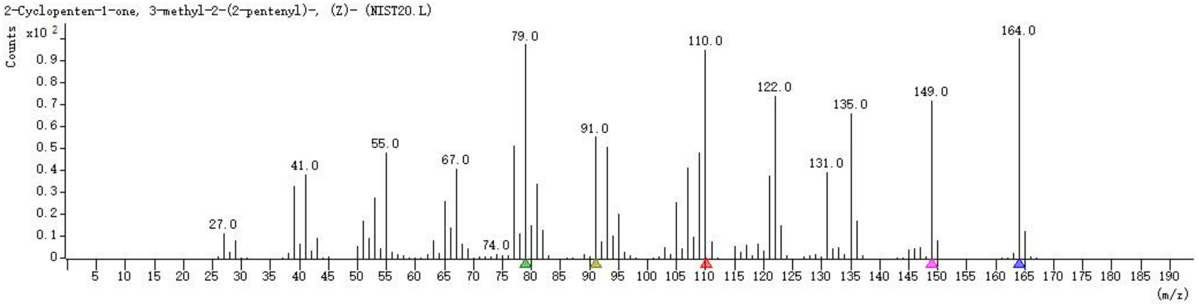
2-Cyclopenten-1-one, 3-methyl-2-(2-pentenyl)-, (Z)- (NIST20.L)
Counts x10 2
79.0
110.0
164.0
122.0
135.0
149.0
91.0
131.0
55.0
67.0
41.0
27.0
74.0
(m/z)

Indole:

A

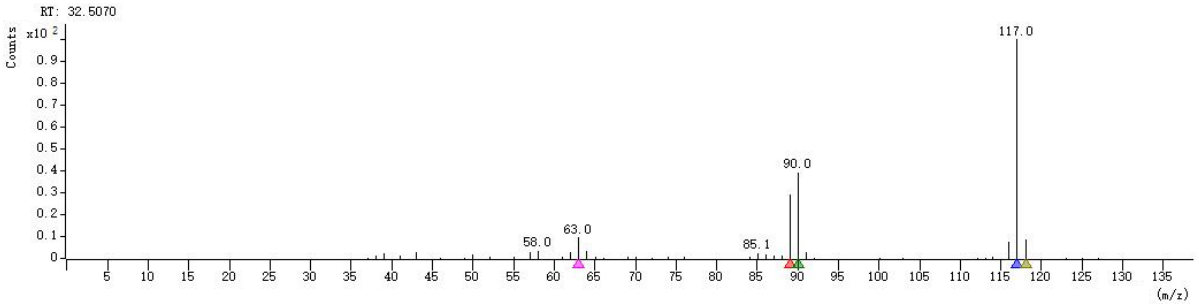
RT: 32.5070
Counts
x10 2
0.9
0.8
0.7
0.6
0.5
0.4
0.3
0.2
0.1
0
117.0
90.0
63.0
58.0
85.1
5
10
15
20
25
30
35
40
45
50
55
60
65
70
75
80
85
90
95
100
105
110
115
120
125
130
135
(m/z)

B

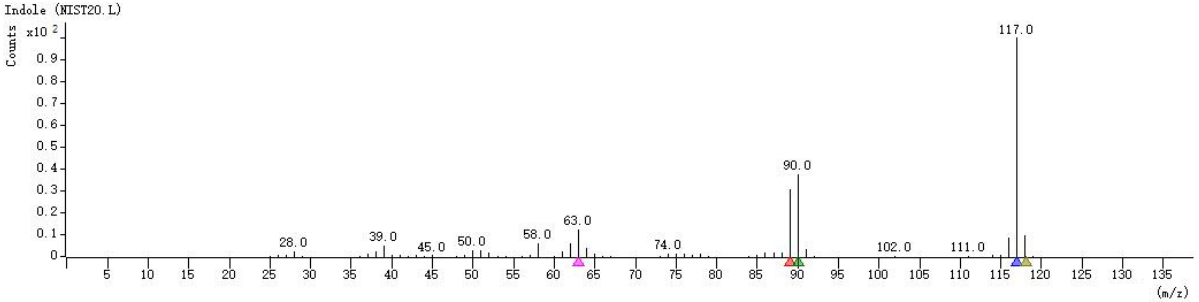
Indole (NIST20.L)
Counts
x10 2
0.9
0.8
0.7
0.6
0.5
0.4
0.3
0.2
0.1
0
117.0
90.0
63.0
58.0
28.0
39.0
45.0
50.0
74.0
102.0
111.0
5
10
15
20
25
30
35
40
45
50
55
60
65
70
75
80
85
90
95
100
105
110
115
120
125
130
135
(m/z)

(Z)-Hexenyl acetate:

A

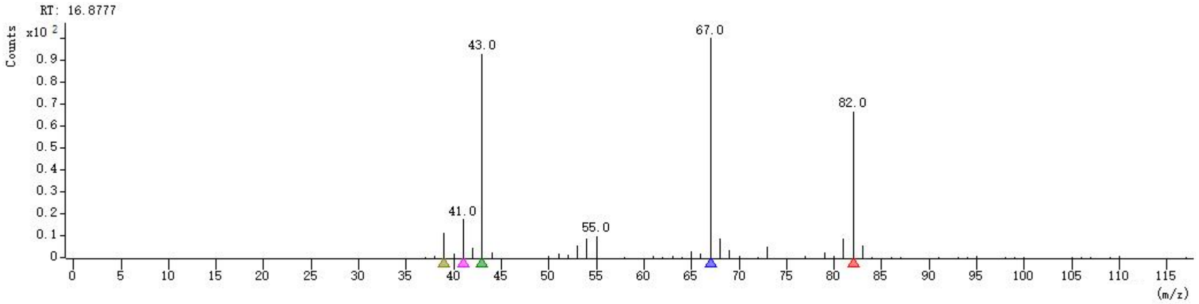

B

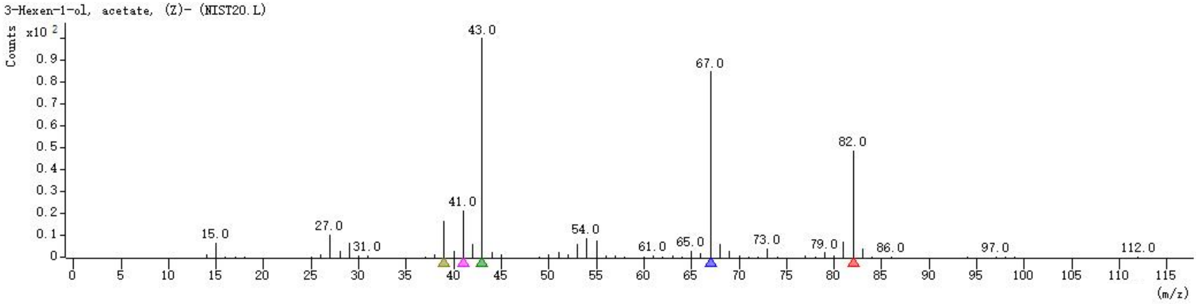

(Z)-3-Hexenyl butanoate:

A

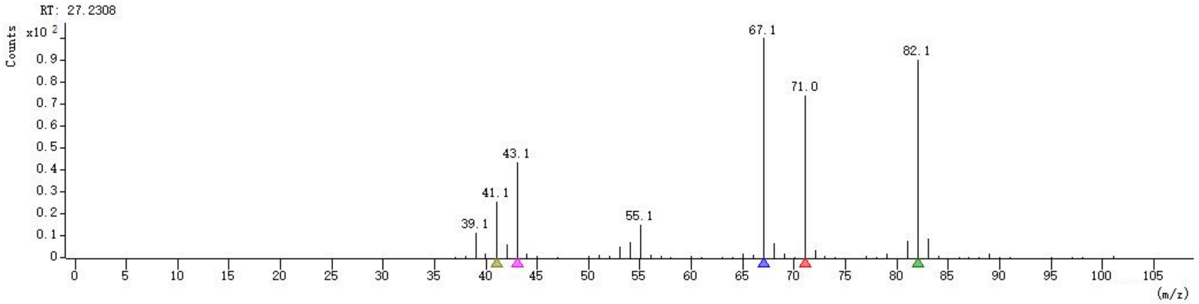

B

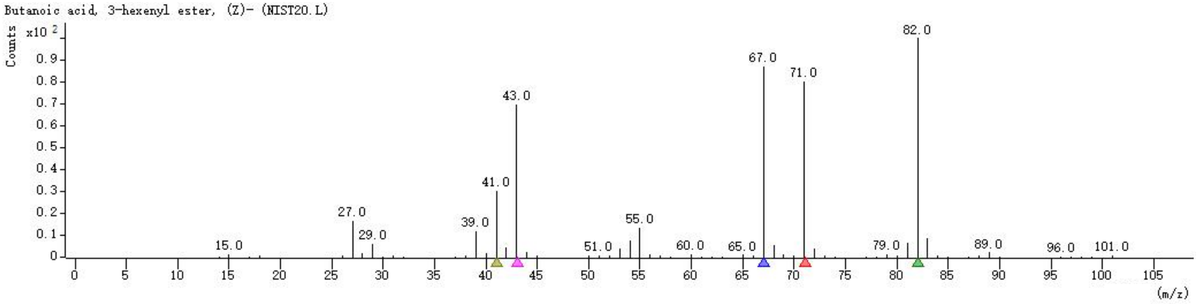

Cis-Linalool oxide(furanoid):

A

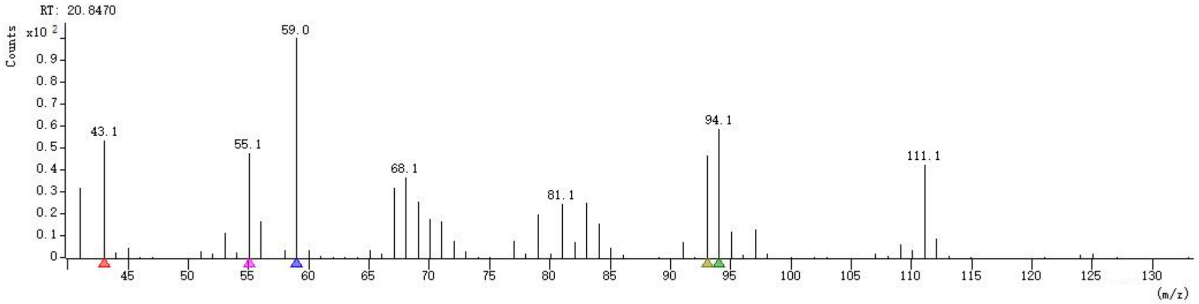

B

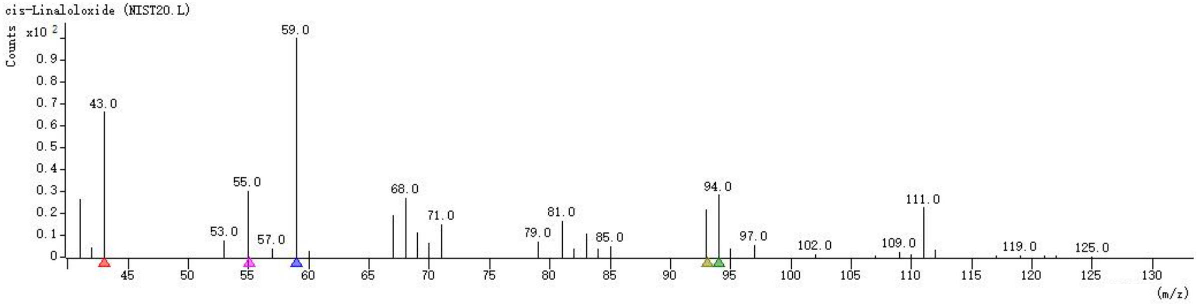

Trans-Linalool oxide(furanoid):

A

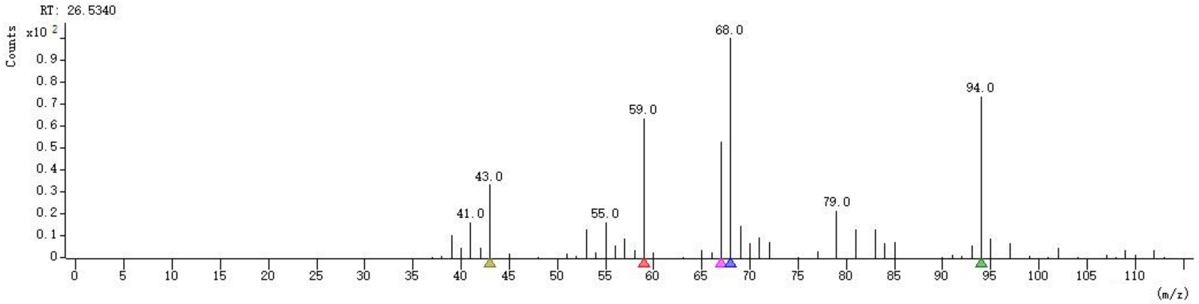

B

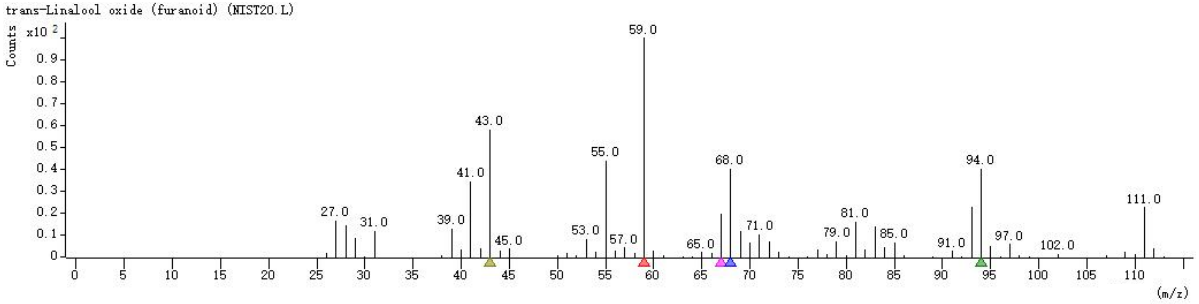

Linalool:

A

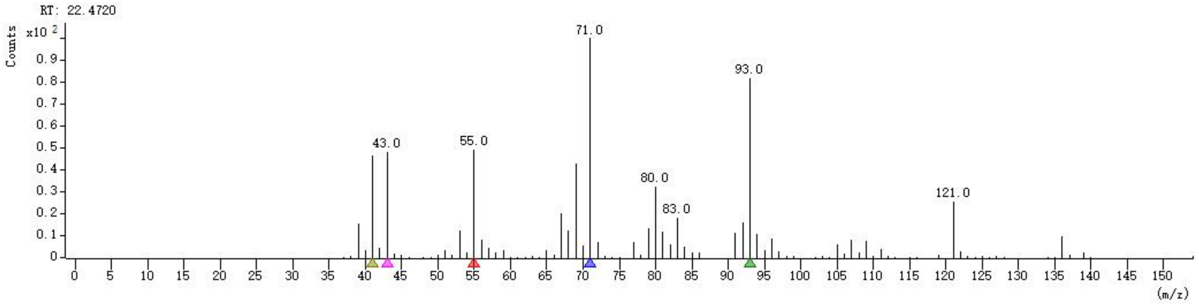

RT: 22.4720
Counts x10 2
71.0
93.0
43.0
55.0
80.0
83.0
121.0
(m/z)

B

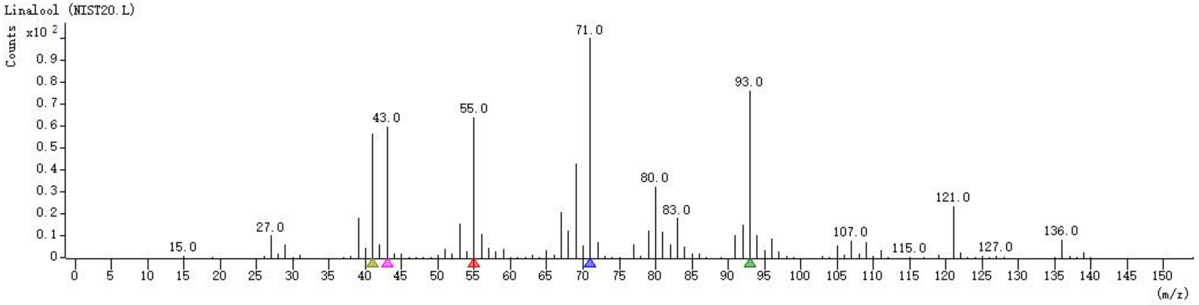

Linalool (NIST20.L)
Counts x10 2
71.0
93.0
43.0
55.0
80.0
83.0
121.0
27.0
15.0
107.0
115.0
127.0
136.0
(m/z)

4-Methyl-3-penten-2-one:

A

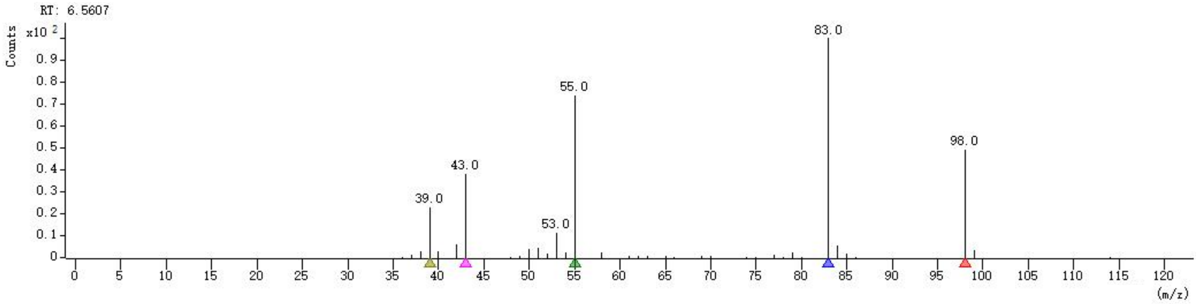

B

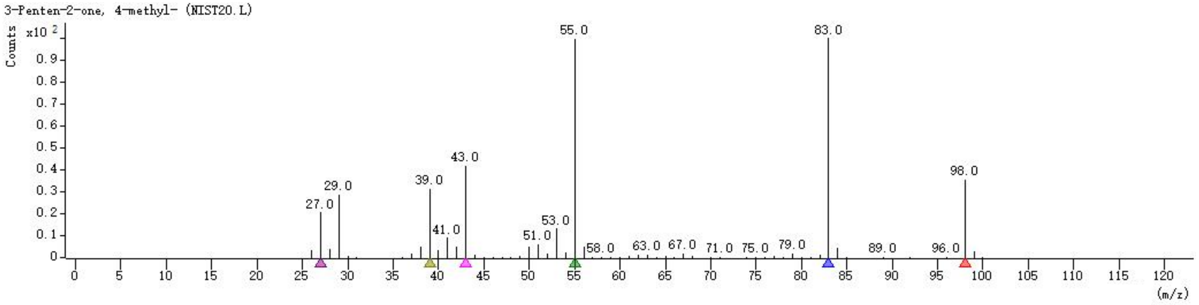

Dimethyl sulfide:

A

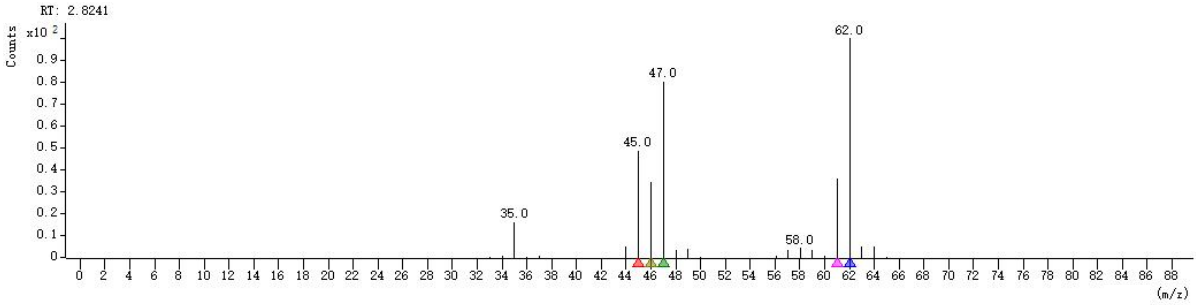

B

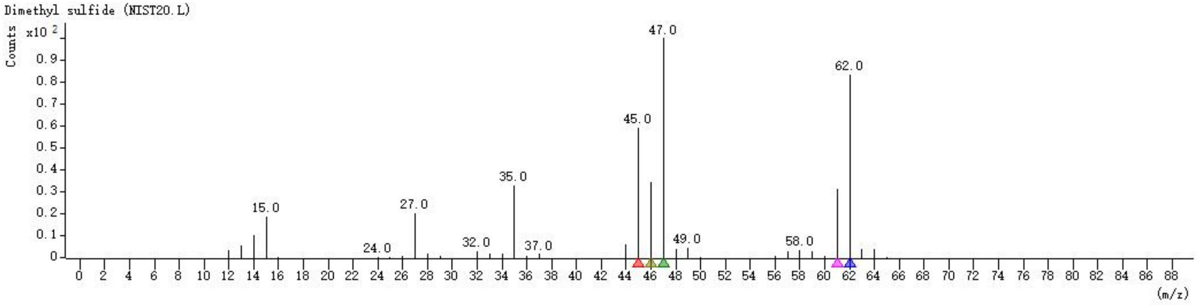

Trans-β-Ionone:

A

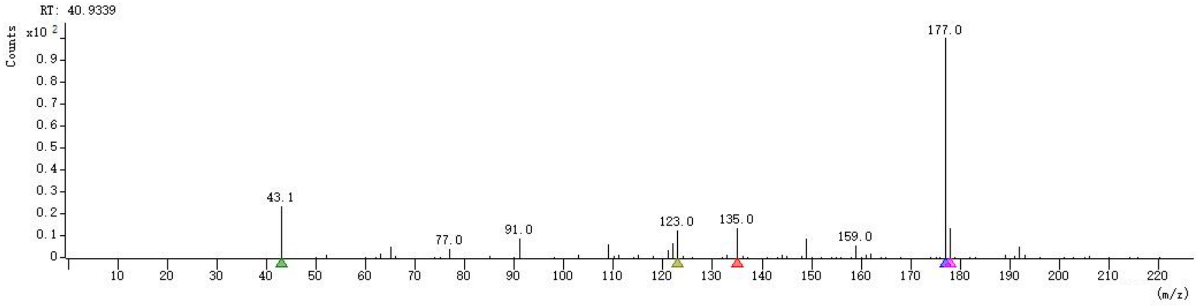

B

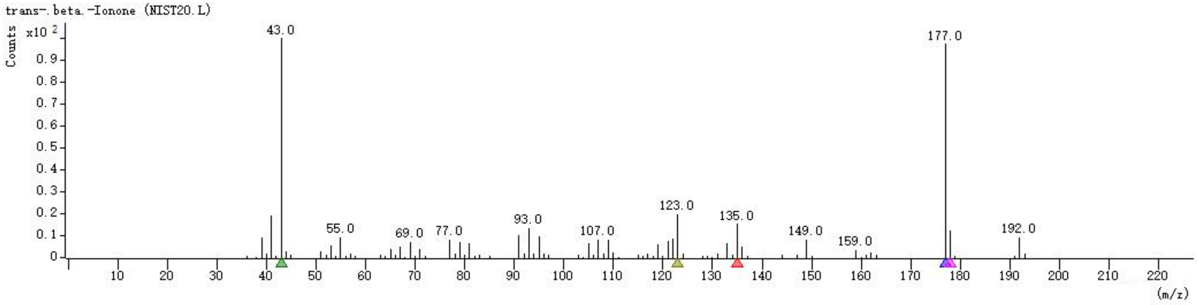

Heptanal:

A

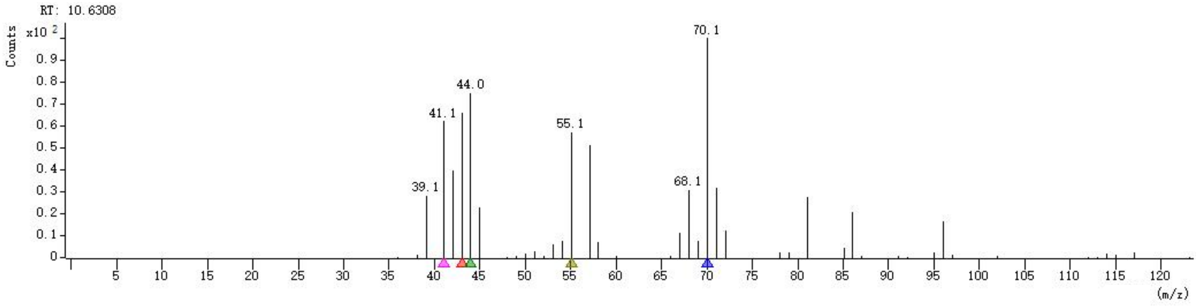
RT: 10.6308
Counts x10 2
0.9
0.8
0.7
0.6
0.5
0.4
0.3
0.2
0.1
0
70.1
44.0
41.1
55.1
39.1
68.1
5 10 15 20 25 30 35 40 45 50 55 60 65 70 75 80 85 90 95 100 105 110 115 120
(m/z)

B

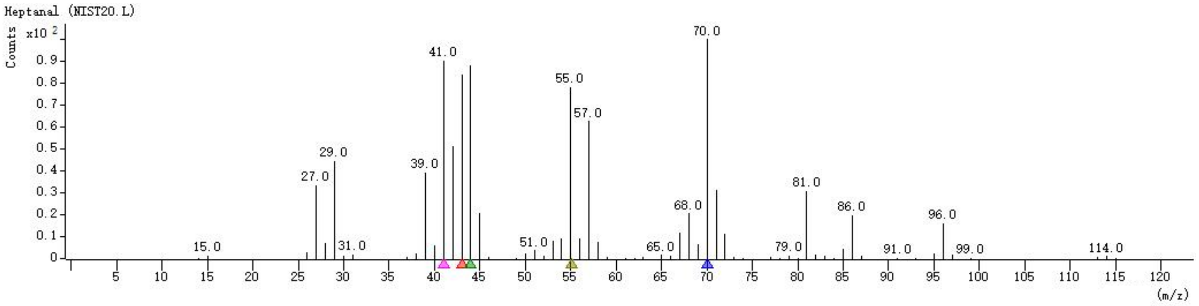
Heptanal (NIST20.L)
Counts x10 2
0.9
0.8
0.7
0.6
0.5
0.4
0.3
0.2
0.1
0
70.0
41.0
55.0
57.0
29.0
27.0
39.0
81.0
86.0
68.0
96.0
15.0
31.0
51.0
65.0
79.0
91.0
99.0
114.0
5 10 15 20 25 30 35 40 45 50 55 60 65 70 75 80 85 90 95 100 105 110 115 120
(m/z)

Nonanal:

A

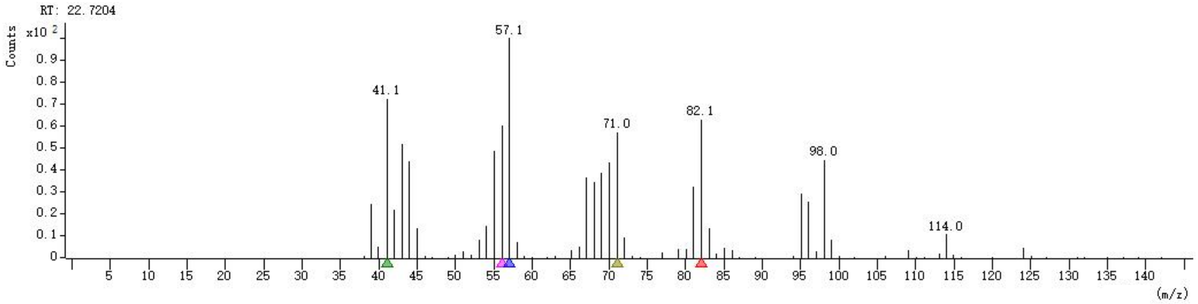
RT: 22.7204
Counts x10 2
57.1
41.1
71.0
82.1
98.0
114.0
(m/z)

B

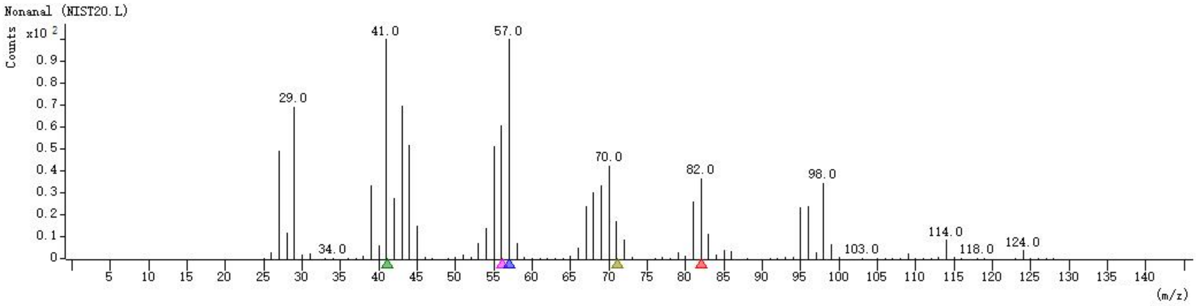
Nonanal (NIST20.L)
Counts x10 2
41.0
57.0
29.0
70.0
82.0
98.0
34.0
114.0
103.0
118.0
124.0
(m/z)

Decanal:

A

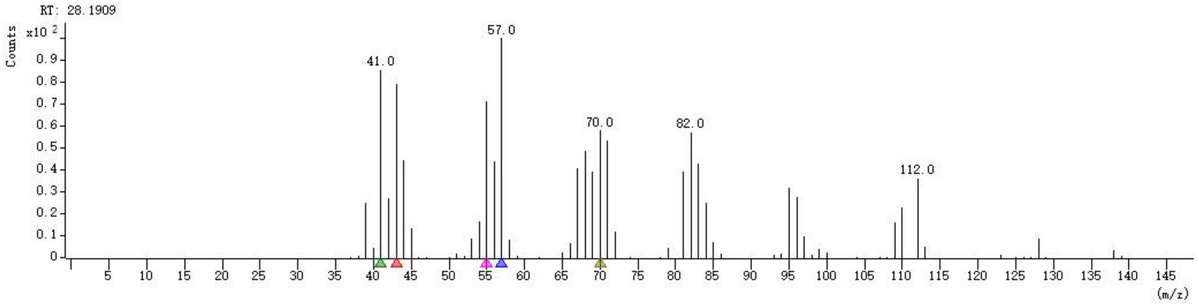
RT: 28.1909
Counts x10 2
41.0
57.0
70.0
82.0
112.0
(m/z)

B

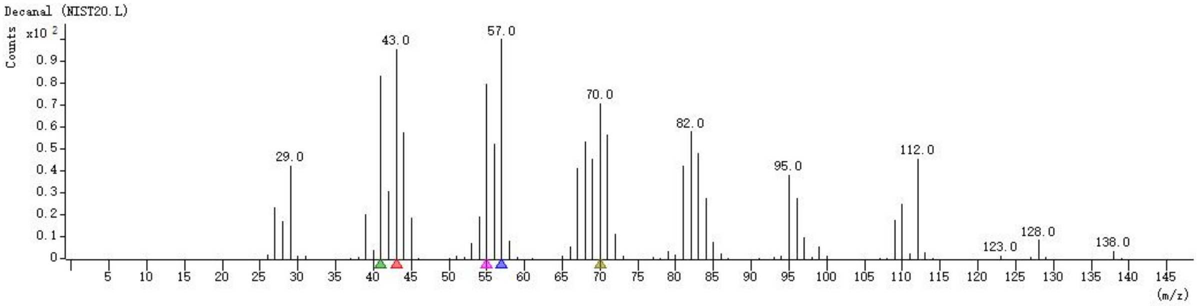
Decanal (NIST20.L)
Counts x10 2
29.0
43.0
57.0
70.0
82.0
95.0
112.0
123.0
128.0
138.0
(m/z)

Octanal:

A

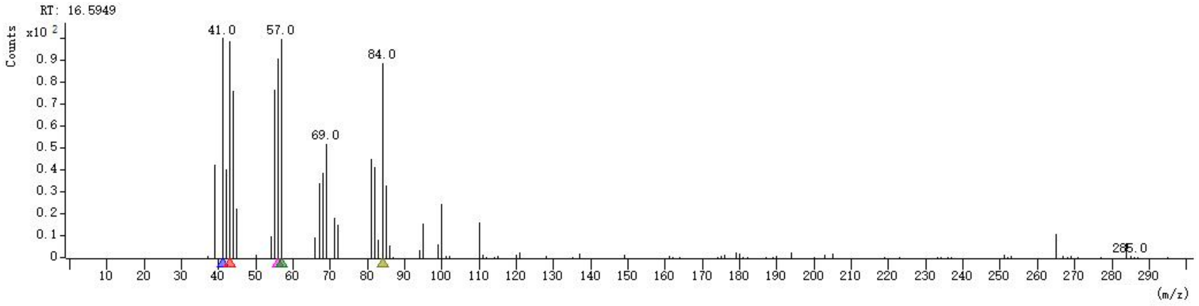
RT: 16.5949
Counts
x10 2
41.0
57.0
69.0
84.0
285.0
(m/z)

B

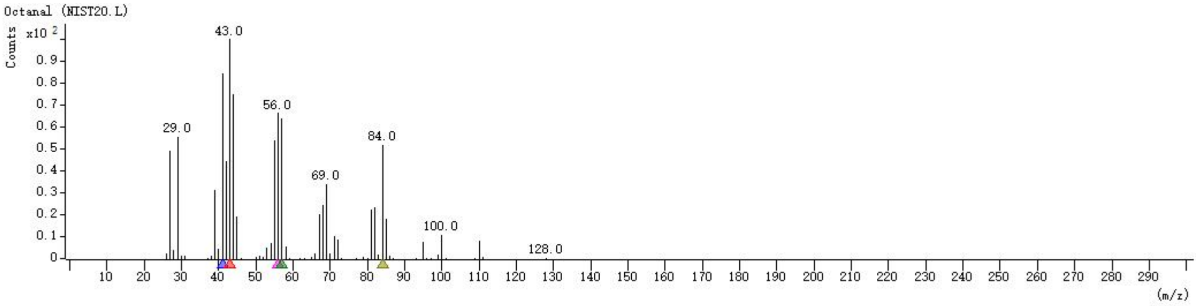
Octanal (NIST20.L)
Counts
x10 2
29.0
43.0
56.0
69.0
84.0
100.0
128.0
(m/z)

3-Methybutanal:

A

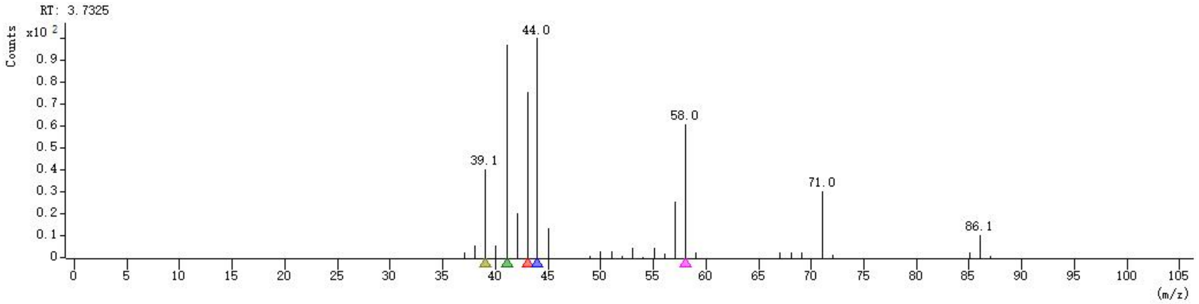

B

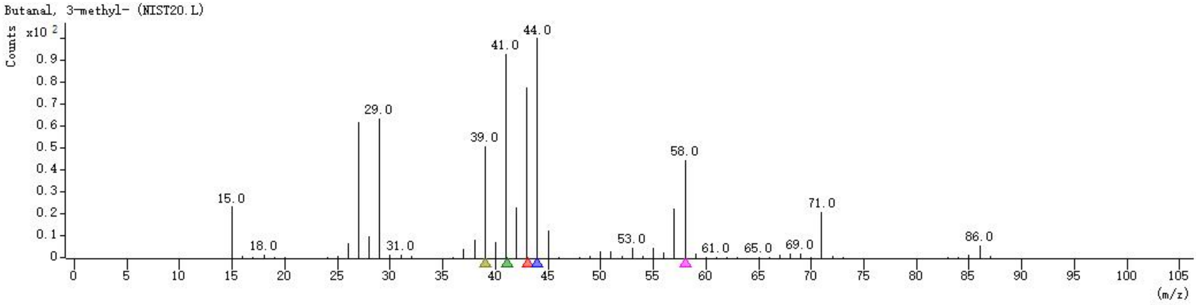

Pentanal:

A

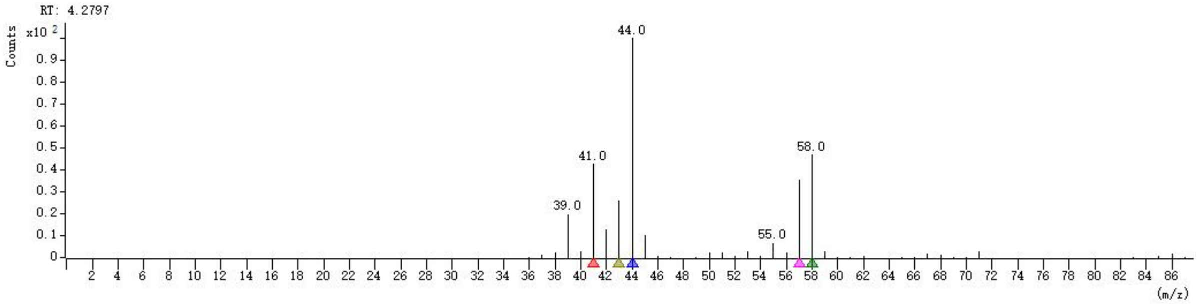
RT: 4.2797
Counts x10 2
(m/z)
44.0
58.0
41.0
39.0
55.0

B

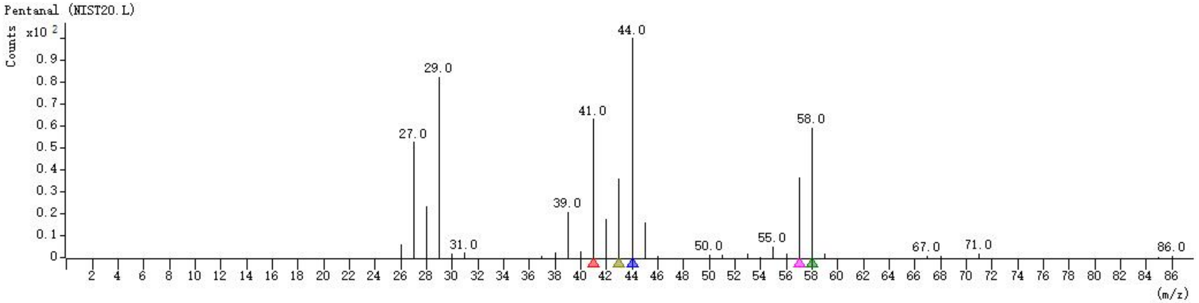
Pentanal (NIST20.L)
Counts x10 2
(m/z)
44.0
29.0
27.0
41.0
58.0
39.0
31.0
50.0
55.0
67.0
71.0
86.0

2-Pentylfuran:

A

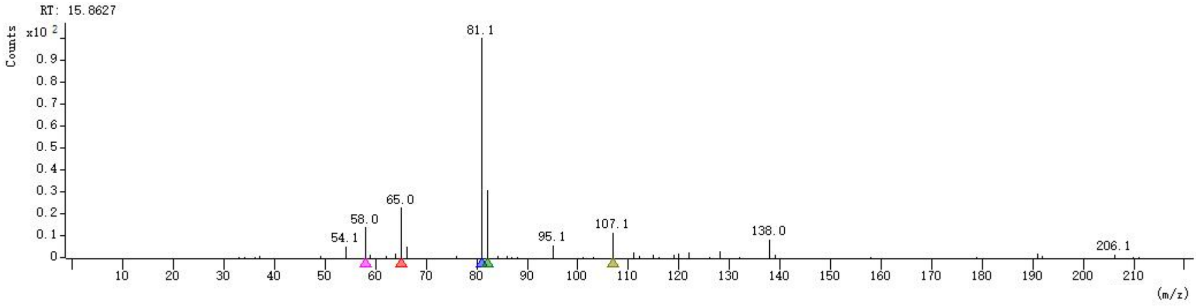

B

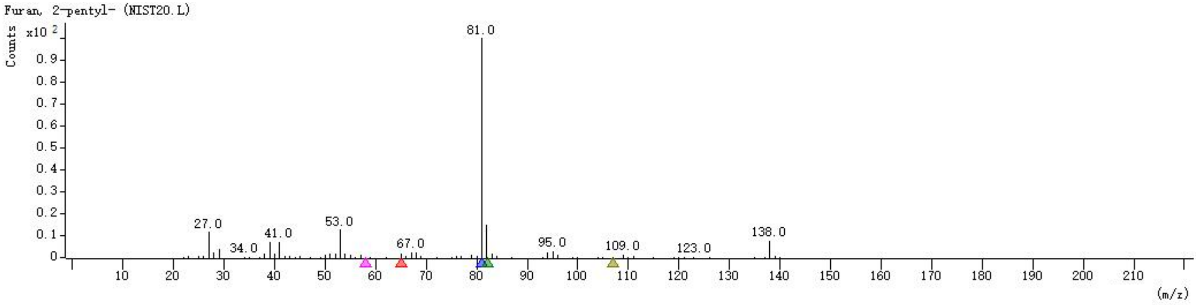

Geraniol:

A

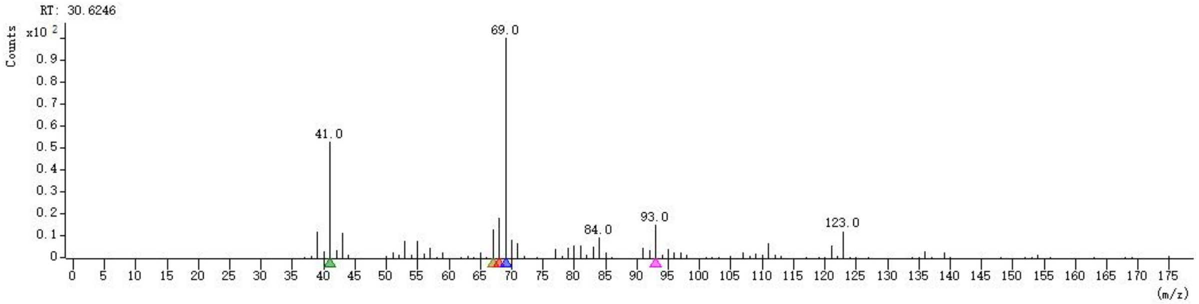
RT: 30.6246
Counts x10 2
69.0
41.0
84.0
93.0
123.0
(m/z)

B

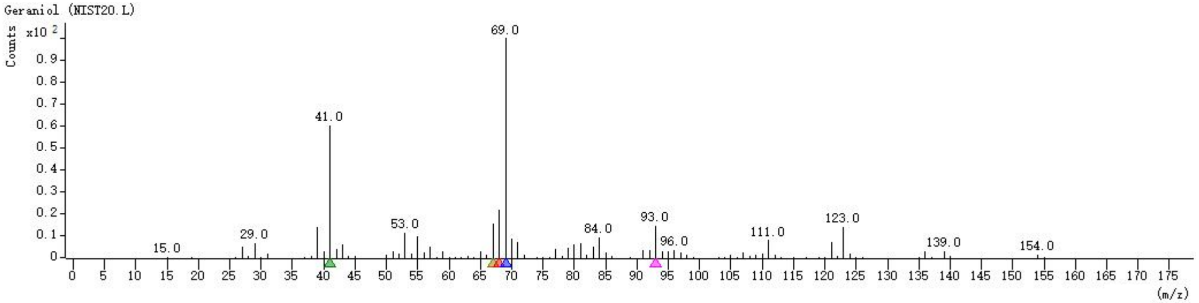
Geraniol (NIST20.L)
Counts x10 2
69.0
41.0
15.0
29.0
53.0
84.0
93.0
96.0
111.0
123.0
139.0
154.0
(m/z)

Hexanal:

A

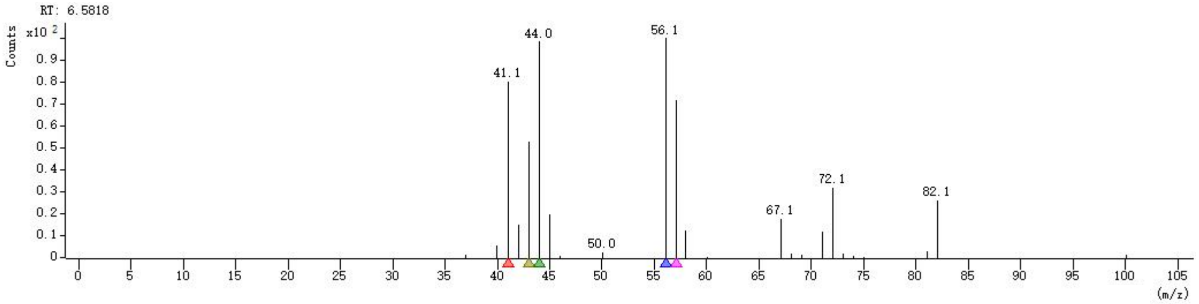
RT: 6.5818
Counts x10 2
44.0
56.1
41.1
67.1
72.1
82.1
50.0
(m/z)

B

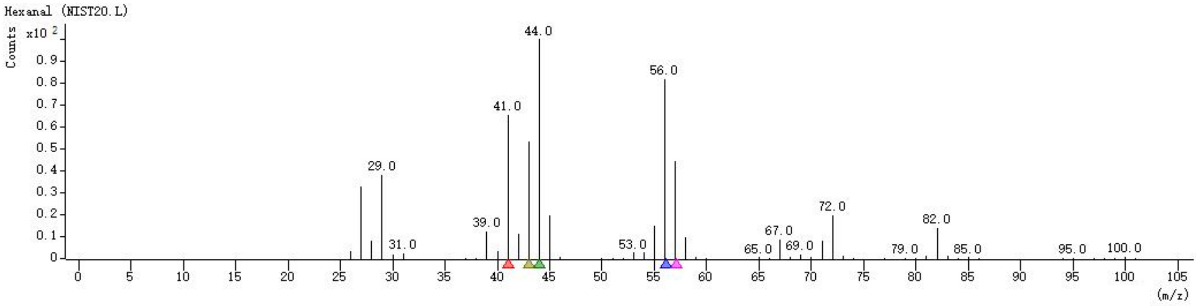
Hexanal (NIST20.L)
Counts x10 2
44.0
56.0
41.0
29.0
31.0
39.0
53.0
65.0
67.0
69.0
72.0
79.0
82.0
85.0
95.0
100.0
(m/z)

2,2,6-Trimethylcycolhexanone:

A

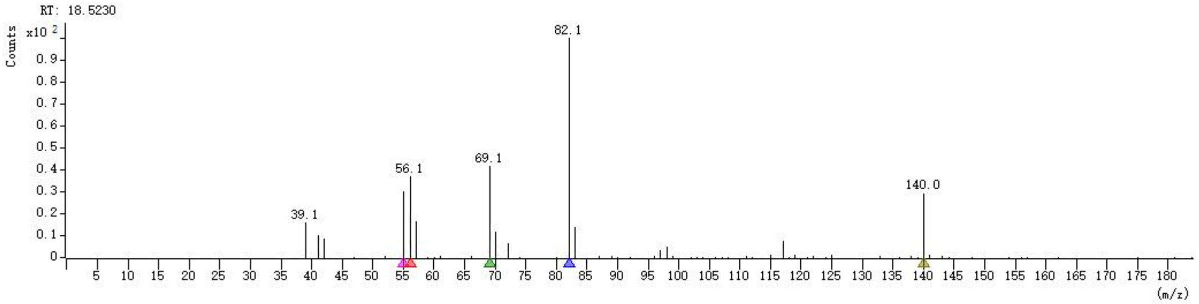

B

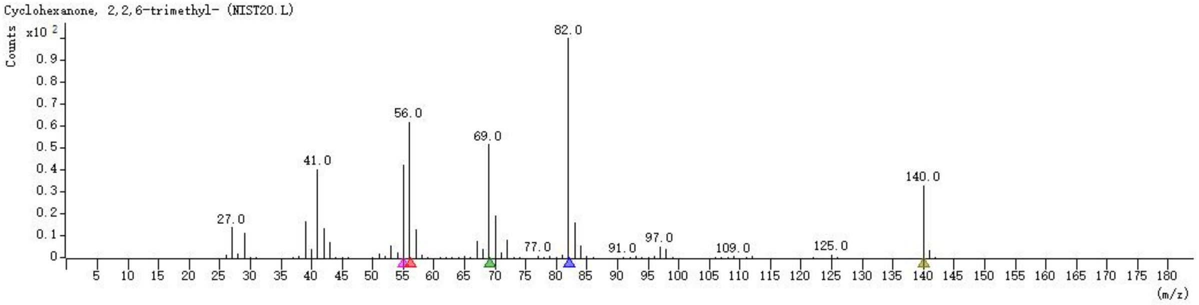

1-Hexanol:

A

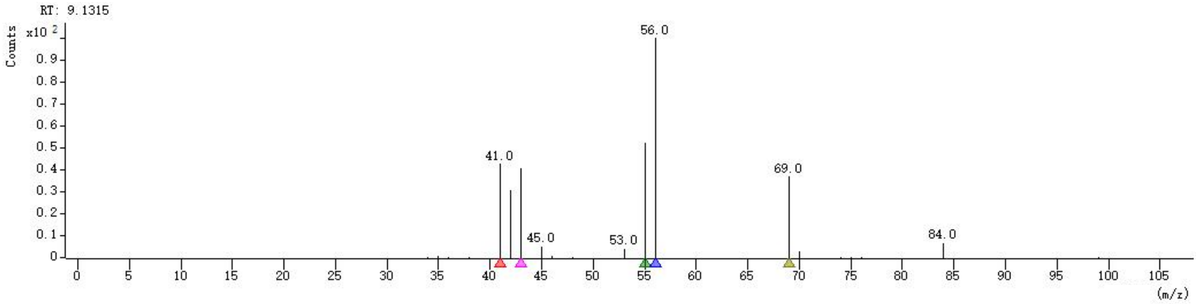
RT: 9.1315
Counts x10 2
0.9
0.8
0.7
0.6
0.5
0.4
0.3
0.2
0.1
0
56.0
41.0
69.0
45.0
53.0
84.0
0 5 10 15 20 25 30 35 40 45 50 55 60 65 70 75 80 85 90 95 100 105
(m/z)

B

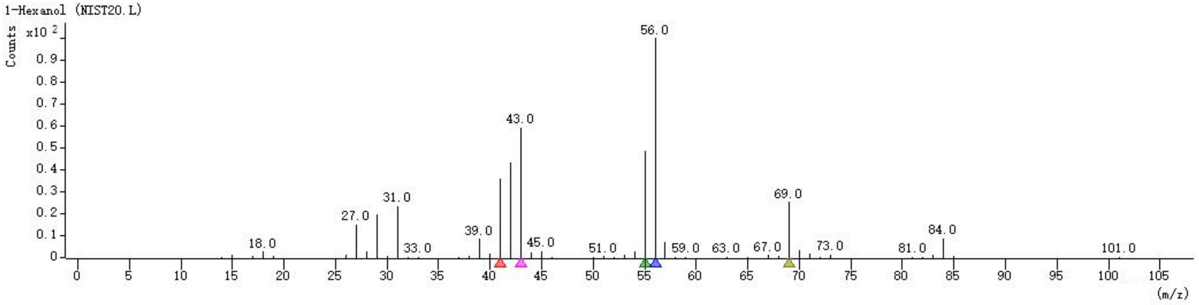
1-Hexanol (NIST20.L)
Counts x10 2
0.9
0.8
0.7
0.6
0.5
0.4
0.3
0.2
0.1
0
56.0
43.0
69.0
31.0
27.0
39.0
84.0
18.0
33.0
45.0
51.0
59.0
63.0
67.0
73.0
81.0
101.0
0 5 10 15 20 25 30 35 40 45 50 55 60 65 70 75 80 85 90 95 100 105
(m/z)

Phenylethyl Alcohol:

A

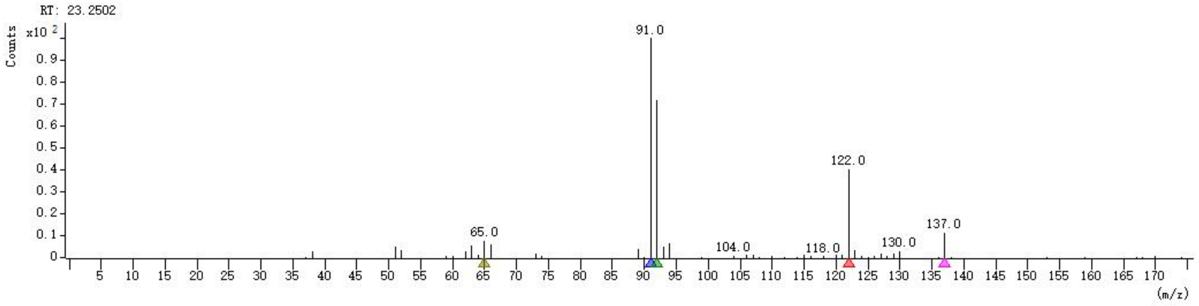

B

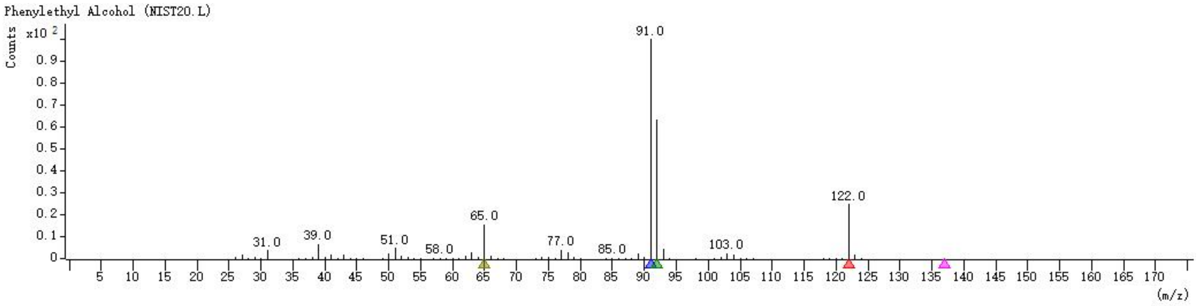

Limonene:

A

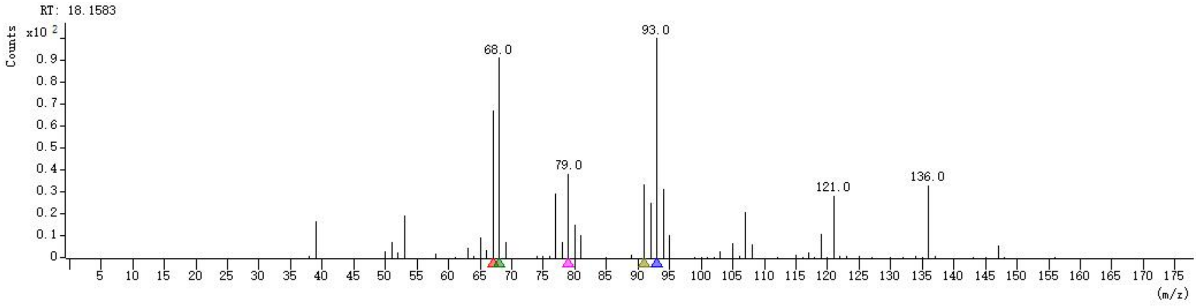
RT: 18.1583
Counts x10 2
68.0
93.0
79.0
121.0
136.0
(m/z)

B

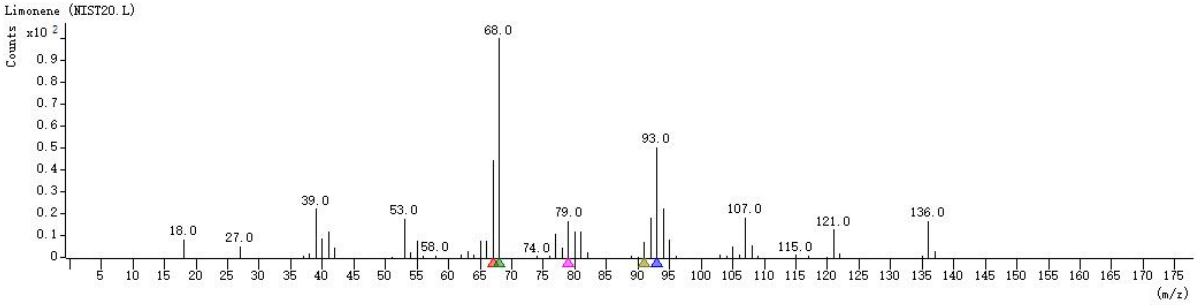
Limonene (NIST20.L)
Counts x10 2
68.0
93.0
18.0
27.0
39.0
53.0
58.0
74.0
79.0
107.0
115.0
121.0
136.0
(m/z)

2-Methylpropanal:

A

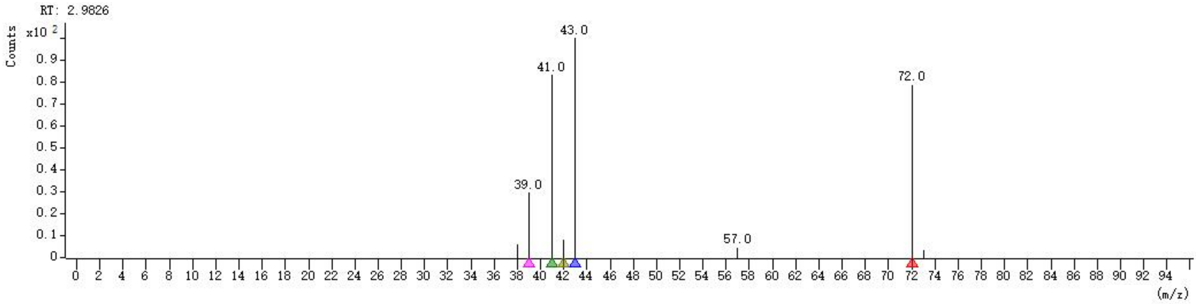

B

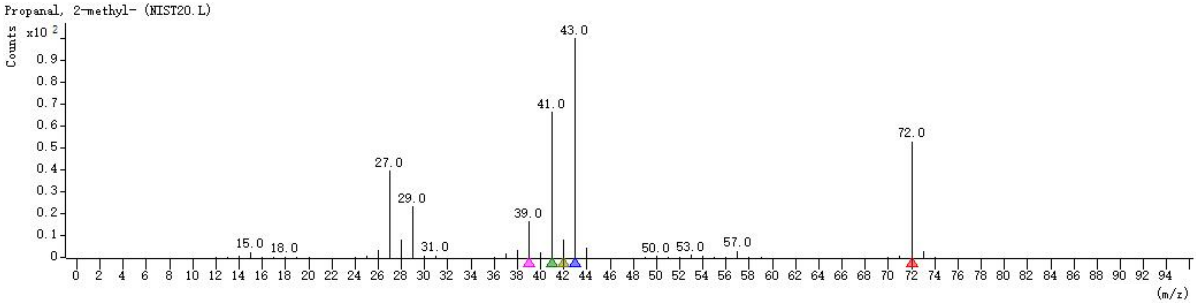

Methyl salicylate:

A

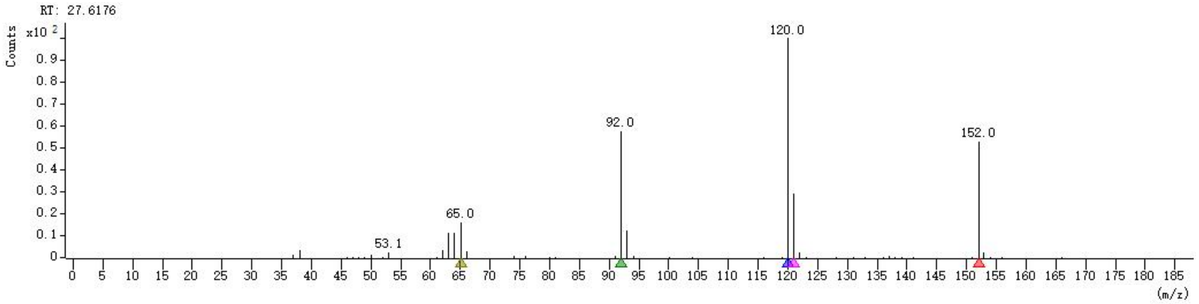

B

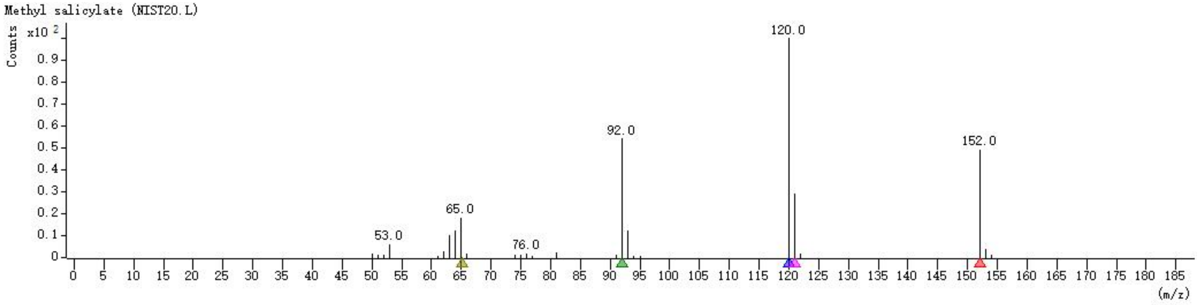

2-Heptanone:

A

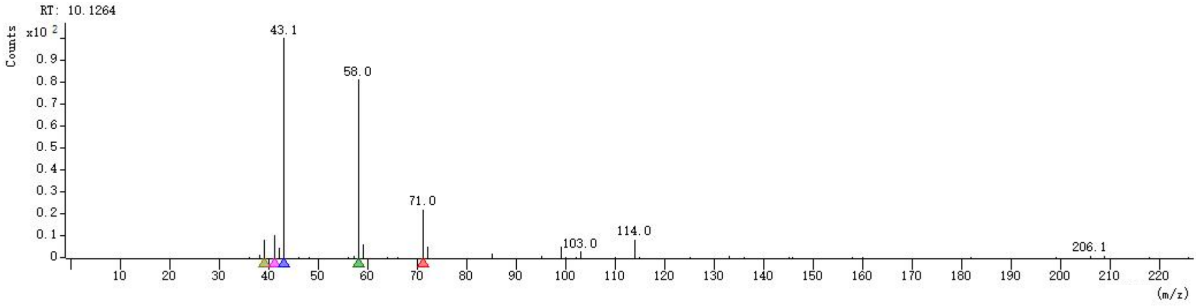

B

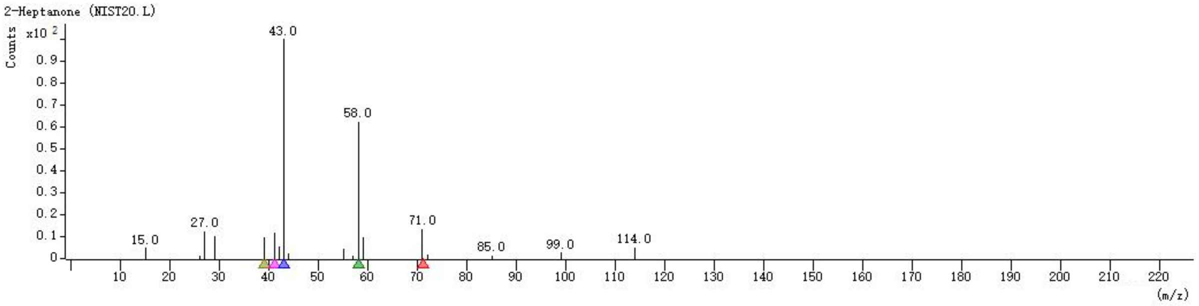

Figure S2. EI mass spectra of 26 key aroma compounds and the corresponding reference spectrum from the NIST20 spectral library. A: EI mass spectrum of the compounds in the sample; B: EI mass spectrum of the compounds matched against the NIST20 spectral library.
